# From knowledge landscapes to network mechanisms: charting regulated cell death pathways in ALS

**DOI:** 10.3389/fnagi.2026.1742805

**Published:** 2026-01-30

**Authors:** Jingxuan Zhang, Zilin Zhao, Tianyue Xiang, Da Teng, Hejia Wan, Qiong Zhang, Xiaohui Liu

**Affiliations:** 1 First Clinical Medical College, Henan University of Chinese Medicine, Zhengzhou, China; 2 School of Nursing (Nursing School of Smart Healthcare Industry), Henan University of Chinese Medicine, Zhengzhou, China

**Keywords:** amyotrophic lateral sclerosis, autophagy, bibliometric analysis, bioinformatics, ferroptosis, immunometabolism, mitochondrial dysfunction, neuroinflammation

## Abstract

**Objective:**

To map the research landscape linking amyotrophic lateral sclerosis (ALS) with regulated cell death (RCD) and to integrate bibliometric trends with bioinformatics evidence to identify convergent mechanisms and actionable targets.

**Methods:**

Web of Science Core Collection, PubMed, and Scopus were searched for 2005–2024 (English; Article/Review). After merging and de-duplication, 6,272 records were analyzed using CiteSpace, VOSviewer, and bibliometrix to evaluate publication trends, collaboration, co-citation structure, and keyword evolution. In parallel, ALS-related genes were intersected with apoptosis-, ferroptosis-, and pyroptosis-associated gene sets. Shared targets were used to construct PPI networks, identify core modules and hub genes, and perform GO/KEGG enrichment analyses.

**Results:**

Publications and citations increased steadily with a clear rise after 2015. The field is anchored by the USA and shows rapidly growing contributions from Asia and Europe. Keyword evolution indicates a shift from “oxidative stress/apoptosis” toward multi-pathway RCD, with prominent recent bursts in ferroptosis, pyroptosis, necroptosis, and autophagy/mitophagy, alongside persistent themes in motor-neuron degeneration, mitochondria, and neuro-inflammation. Bio-informatics results showed substantial genetic overlap between ALS and RCD modalities. Hub-gene analysis highlighted TP53, AKT1, STAT3, MYC, RELA, EP300, CREBBP, JUN, HSP90AA1, and MAPK3 as central nodes. Enrichment analyses implicated FoxO, HIF-1, and lipid-related pathways, and GO terms related to chemical/oxidative stress responses and autophagy regulation.

**Conclusion:**

ALS–cell death research is consolidating around interconnected RCD programs. Integrated bibliometric and bioinformatics evidence supports an immunometabolic convergence involving ferroptosis–inflammation–autophagy signaling, providing a focused set of candidate pathways and hub targets for mechanistic validation and translation.

## 1 Introduction

Cell death is fundamental to tissue homeostasis and stress adaptation. In contemporary biology, it encompasses well-established regulated programs—such as apoptosis, necroptosis, pyroptosis, and autophagy-associated cell death—as well as several newly described modalities that have rapidly expanded the regulated cell death (RCD) landscape over the past decade (He et al., 2025; Moujalled et al., 2021). Among these, ferroptosis represents an iron-dependent, lipid-peroxidation–driven death mode that is molecularly and morphologically distinct from apoptosis and necrosis (Stockwell, 2022; Yan et al., 2021). Additional emerging modalities include cuproptosis, in which copper binding to lipoylated tricarboxylic-acid–cycle enzymes induces proteotoxic aggregation and metabolic collapse (Tsvetkov et al., 2022), and disulfidptosis, which occurs under SLC7A11-high and glucose-stress conditions and features catastrophic disulfide stress with F-actin cytoskeletal failure (Liu et al., 2023). In neurodegenerative diseases, these regulated death programs intersect with oxidative stress, mitochondrial dysfunction, lipid peroxidation, and neuroinflammation, shaping a mechanistic spectrum that involves both neurons and glia (Moujalled et al., 2021; Jiang et al., 2015).

Amyotrophic lateral sclerosis (ALS) is a fatal neurodegenerative disorder characterized by progressive upper and lower motor neuron degeneration, clinical heterogeneity, insidious onset, and rapid progression. Most cases are sporadic, with 5–10% familial; molecular pathology commonly involves TDP-43 proteinopathy, SOD1 and FUS mutations, and C9orf72 repeat expansion (Brown and Al-Chalabi, 2017; Hardiman et al., 2017). From a population perspective, analyses of GBD 2019 and recent systematic reviews estimate an age-standardized incidence near 0.8 per 100,000 person-years and prevalence roughly 3–9 per 100,000, with marked geographic variation (Liu et al., 2025; Park et al., 2022; Wolfson et al., 2023). Although riluzole and edaravone are foundational treatments, their benefits remain modest. The therapeutic landscape is evolving, with recent approvals such as oral edaravone in 2022 and tofersen for SOD1-mutant ALS in 2023, the first gene-targeting therapy for the disease (Blair, 2023). However, these advances are tempered by setbacks, including the 2024 market withdrawal of sodium phenylbutyrate–taurursodiol (Relyvrio) after a Phase 3 trial failed to confirm its efficacy (Bradford and Rodgers, 2024). Current research focuses heavily on next-generation approaches like antisense oligonucleotides, gene therapies, and stem cell-based treatments, though a unifying framework to halt disease progression across all patient populations is still needed (Erdi-Krausz and Shaw, 2025).

Against this background, “cell death” has become a high-frequency yet conceptually diffuse theme in ALS research: ferroptosis, cuproptosis, disulfidptosis, and classical death programs often intertwine, terminology continues to evolve, and subfields proliferate—making it difficult to synthesize mechanistic priorities and track frontiers in a rapidly growing literature. Bibliometrics offers a scalable way to integrate large corpora using co-occurrence networks, burst-term detection, and temporal evolution analyses, enabling identification of core countries/institutions/authors, knowledge bases, and emerging topics (Chen, 2006; van Eck and Waltman, 2010; Aria and Cuccurullo, 2017). Accordingly, this study employs bibliometric methods to map the spatiotemporal landscape, thematic structure, and research frontiers at the “ALS & cell death” interface; to position distinct death programs along putative ALS mechanistic chains and therapeutic targets; and to provide actionable directions for mechanistic validation and clinical translation. We combined the respective advantages of three bibliometric software tools to complete the bibliometric analysis part of this study’s topic (Supplementary Figure 1).

## 2 Materials and methods

### 2.1 Data collection

We queried the Web of Science Core Collection (WoSCC), PubMed, and Scopus because its stable field tags, export formats, and documented retrieval quality facilitate reproducible bibliometric workflows. In line with recommended good practice for database selection, WoSCC is widely used in science-mapping and performs reliably for structured, query-based searches (Gusenbauer and Haddaway, 2020).

Our Topic search combined generic and specific cell-death terms with ALS synonyms across three databases:

- WoSCC (Topic Search, TS): TS = (“cell death” OR “programmed cell death” OR apoptosis OR necrosis OR pyroptosis OR ferroptosis OR “iron death” OR “copper death” OR cuproptosis OR “ammonia death” OR PANoptosis) AND TS = (“amyotrophic lateral sclerosis” OR ALS OR “Lou Gehrig’s disease” OR “progressive muscle atrophy”).
- PubMed (All fields): (cell death OR programmed cell death OR apoptosis OR necrosis OR pyroptosis OR ferroptosis OR iron death OR copper death OR cuproptosis OR ammonia death OR PANoptosis) AND (amyotrophic lateral sclerosis OR Lou Gehrig’s disease OR progressive muscle atrophy OR ALS).
- Scopus (TITLE-ABS-KEY): TITLE-ABS-KEY (cell death OR programmed cell death OR apoptosis OR necrosis OR pyroptosis OR ferroptosis OR iron death OR copper death OR cuproptosis OR ammonia death OR PANoptosis) AND TITLE-ABS-KEY (amyotrophic lateral sclerosis OR Lou Gehrig’s disease OR progressive muscle atrophy OR ALS).

The time span was 2005–2024; document types were limited to Article and Review; the language was English.

The time span was 2005–2024, document types were limited to Article and Review, and the language was English. We exported the “Full Record and Cited References” in plain-text format for downstream tools.

Records from the three databases were merged, and duplicates were removed using the Data Conversion and Processing Utility within CiteSpace.

**Figure 1:**
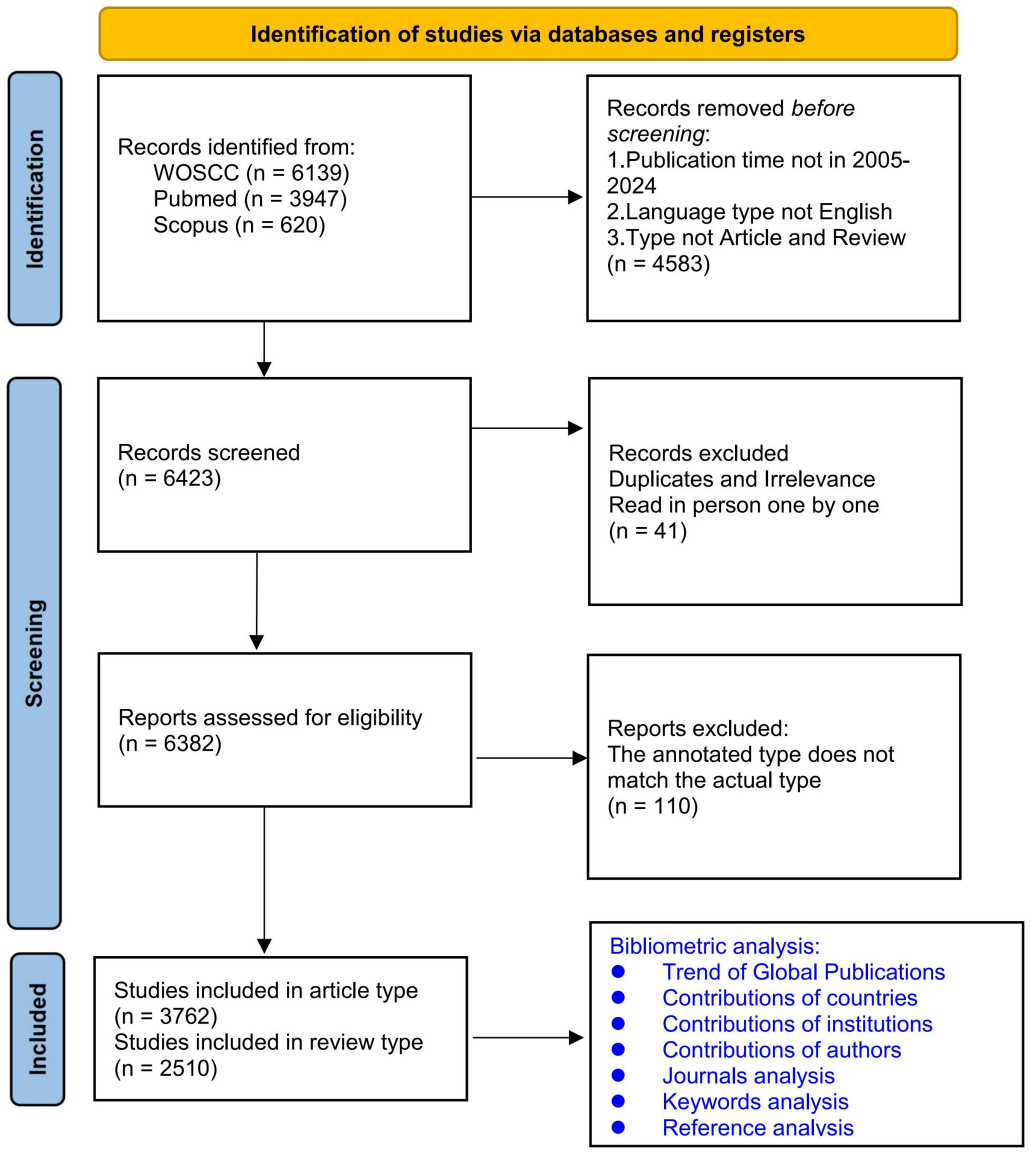
Flowchart of inclusion and exclusion (PRISMA-style).

A PRISMA-style flow (Figure 1) was used to transparently report identification, screening, eligibility, and inclusion, improving interpretability of the selection process even for non-systematic mapping studies (Page et al., 2021). To anchor results in the ALS and neuroscience domain, we also cross-checked our retrieval strategy against two recent domain-adjacent bibliometric studies on ALS gene editing (Wan et al., 2024b).

### 2.2 Data analysis

Downloaded records were harmonized for core fields (authors, title, source, keywords, affiliations, countries, references) and analyzed using a complementary toolkit: CiteSpace for co-citation networks, burst detection, clustering, and timeline views (Chen, 2006); VOSviewer for distance-based mapping and overlay/density visualizations of countries/regions, institutions, authors, journals, and keywords (van Eck and Waltman, 2010); and the R package bibliometrix for descriptive indicators and science-mapping routines, including thematic mapping and historiographic overlays (Aria and Cuccurullo, 2017). Networks considered the classical families of indicators in bibliometrics—citation/co-citation (Garfield, 1955; Small, 1973), bibliographic coupling, and co-word structures. CiteSpace parameters followed widely used defaults for interpretability in large corpora (time-slicing by year; Pathfinder pruning; clustering with LLR; modularity Q and silhouette S for cluster quality; Kleinberg’s burst detection), while VOSviewer used association-strength normalization for layout optimization. Reporting followed recognized guidelines for transparency in bibliometric reviews (Donthu et al., 2021). We appropriately adjusted the bibliometric analysis parameters in this study based on the parameters used in previous publications by our team (Wan et al., 2024a; Wan et al., 2024b).

We acknowledge that bibliometric software packages implement different counting rules and analytical algorithms (e.g., full vs. fractional counting, normalization strategies, and handling of multi-affiliation records), which can lead to discrepancies when analyzing the same indicator across tools. In our analyses, such differences were indeed observed, particularly for entities with similar publication volumes.

To ensure interpretability and methodological consistency, we therefore applied a predefined priority rule rather than attempting to merge outputs across tools. Specifically, VOSviewer was prioritized for author-, institution-, and country-level analyses, as these indicators primarily reflect production and collaboration structures and benefit from VOSviewer’s association-strength normalization and stable network layouts. CiteSpace and bibliometrix/R were prioritized for keyword-related analyses, including burst detection and thematic evolution, where their algorithms are more appropriate for identifying research fronts and temporal dynamics.

Because these indicators capture fundamentally different dimensions of the literature and are generated under distinct algorithmic assumptions, results from different tools were not combined for the same indicator. When discrepancies occurred, the results generated under the pre-specified priority tool were reported, with clear attribution to the software used, to maintain transparency and reproducibility.

### 2.3 Parameter settings (bibliometrics)

CiteSpace: Time slicing = 1 year; Pathfinder pruning; LLR (Log-Likelihood Ratio) algorithm for clustering; Modularity (Q) and Silhouette (S) scores reported for cluster quality; Kleinberg’s burst detection algorithm.

At that time, cluster analysis was used (usually for Co-cited keyword analysis, suitable for the following parameter settings)

- Selection criteria: g-index (k = 25), LR*F* = 3.0, L/N = 10, LBY = 5, e = 1.0
- Network: Number of nodes *N* = 813, number of edges F = 3,313 (※density = 0.01)
- Largest connected component: 813 (100%)
- Labeled nodes: 1.0%
- Pruning: MST
- Modularity Q = 0.4018
- Weighted average silhouette coefficient S = 0.7203
- Adjusted harmonic mean (Q, S) = 0.5158

Also, cluster analysis was used (usually for Co-cited Literature analysis, suitable for the following parameter settings)

- Timespan:2005–2024 (Slice Length = 1)
- Selection Criteria: g-index (k = 25), LRF = 3.0, L/N = 10, LBY = 5, e = 1.0
- Network: *N* = 1717, E = 8,625(Density = 0.0059)
- Largest 1 CCs:1559(90%)
- Nodes Labeled:1.0%
- Pruning: None
- Modularity Q = 0.721
- Weighted Mean Silhouette S = 0.8799
- Harmonic Mean (Q, S) = 0.7926

VOSviewer: Minimum number of occurrences used to create contribution network; Association strength normalization for mapping; clustering resolution parameter set to default (1.0).

R: Depending on the software package, its internal settings are mainly fine-tuned according to the functions.

When it comes to co-cited documents, co-cited authors, and co-cited journals, because bibliometric software can only process data exported from WOS and cannot clean or merge data, we only use the cleaned data exported from WOSCC to conduct the relevant co-citation analysis. However, the adjustment parameters are the same as the other analysis parameters.

### 2.4 Bioinformatics analysis

To systematically investigate the molecular mechanisms linking Amyotrophic Lateral Sclerosis (ALS) with regulated cell death (RCD), we harnessed public databases to retrieve targets related to ALS and RCD modalities, including apoptosis, ferroptosis, and pyroptosis. The intersection of these gene sets was visualized using Venn diagrams to identify common candidate targets. Subsequently, a protein–protein interaction (PPI) network was constructed utilizing the STRING database and visualized with Cytoscape software (version 3.9.0). To delve deeper into the network topology and identify core regulatory sub-networks, we employed the MCODE and CytoNCA plugins within Cytoscape. Furthermore, to elucidate the biological functions and signaling pathways associated with these shared targets, we employed the R packages “clusterProfiler”, “org.Hs.eg.db”, and ‘ggplot2’ to conduct enrichment analyses across Kyoto Encyclopedia of Genes and Genomes (KEGG) and Gene Ontology (GO) databases. The GO analysis encompassed biological processes (BP), molecular functions (MF), and cellular components (CC), providing a comprehensive functional annotation of the identified targets.

## 3 Results

### 3.1 Search results

The tri-database search (WoSCC, PubMed, and Scopus) and PRISMA-style workflow are shown in Figure 1. Initially, 11,006 records were retrieved across databases. After applying time (2005–2024), language (English), and document-type (Article/Review) filters, 6,423 records remained. Following de-duplication and exclusion of irrelevant items, 6,382 records were included, and finally 6,272 for bibliometric analyses (Figure 1).

### 3.2 Analysis of publishing countries

**Figure 2:**
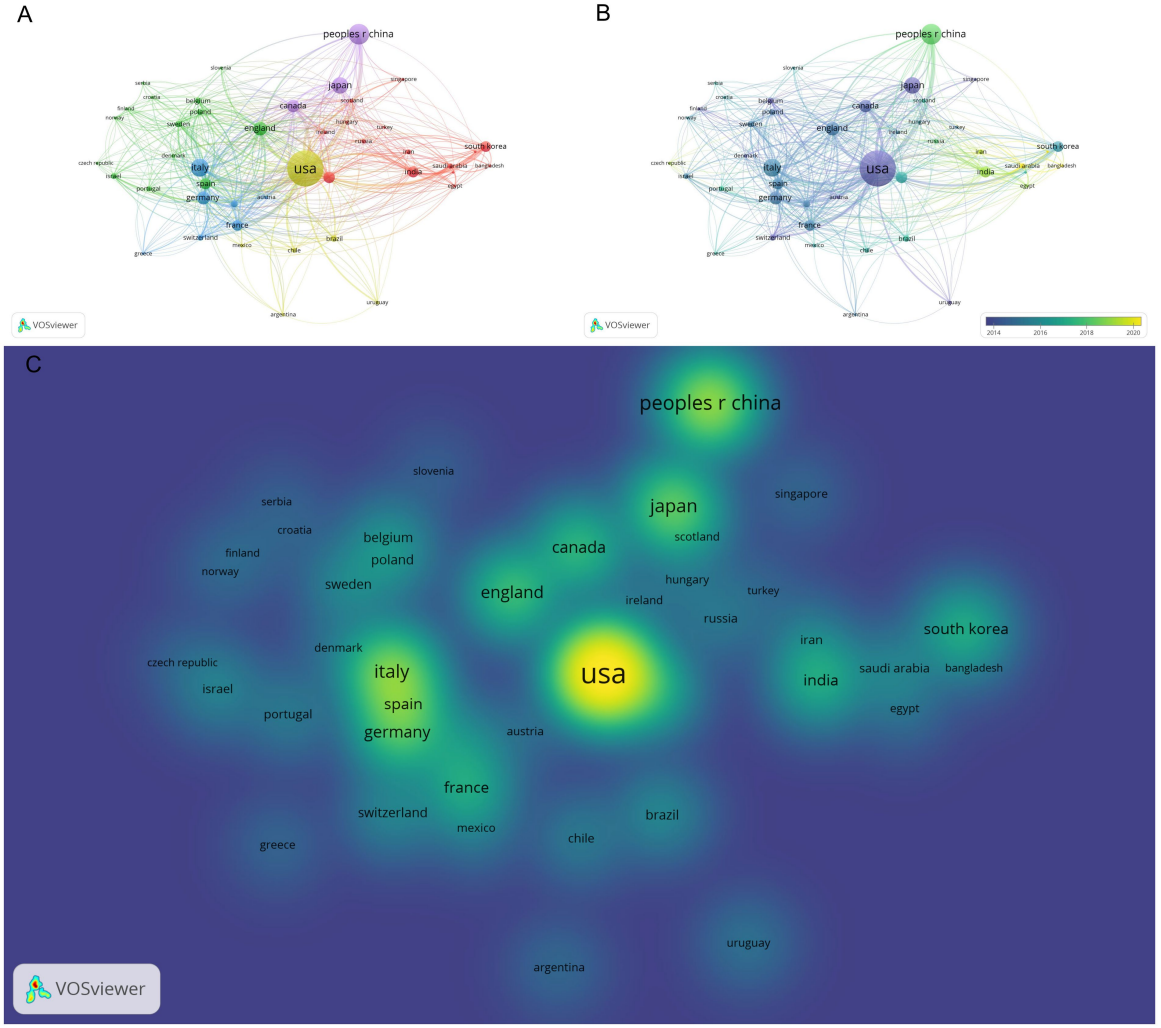
Clustered maps of countries based on publications (the node has a minimum threshold of 10): **(A)** Cluster map of countries based on publications, **(B)** time distribution map of country/region clusters based on publications, **(C)** hotspot map of country/region clusters based on publications.

The Country / Region collaboration map shows a multi-cluster structure anchored by the USA at the network core, linked densely with Europe and the Asia–Pacific (Figure 2A). A time-overlay highlights early leadership by the USA, the UK, and Germany, followed by a marked surge in East Asia, particularly China, Japan, and South Korea (Figure 2B). The density view confirms the USA as the principal hotspot, with strong secondary hubs in China, Japan, the UK, Italy, and Germany (Figure 2C). Quantitatively, the top 20 countries together contributed 5,540 documents and 395,414 citations. The USA leads with 1,640 documents and 148,379 citations, followed by Italy (476, 34,098), the UK (306, 33,445), China (603, 22,419), Germany (268, 21,798), Japan (415, 18,762), Australia (214, 17,856), Canada (262, 17,369), South Korea (198, 15,547), and France (190, 12,597) (Supplementary Table 1; Figures 2A–C).

### 3.3 Analysis of publishing institutions

Institutional collaboration likewise forms a polycentric topology, with Johns Hopkins University, Harvard University, the University of Melbourne, the University of California system, Columbia University, King’s College London, and Massachusetts General Hospital acting as cross-cluster connectors (Supplementary Figure 2a). Time overlays indicate that these leading institutions have maintained consistent output and influence across the study period (Supplementary Figure 2b). Johns Hopkins University ranks first by citations (100 documents; 12,999 citations), followed by Harvard University (70; 7,533), the University of Melbourne (59; 9,878), UC San Diego (56; 9,457), UC San Francisco (33; 8,710), King’s College London (46; 7,327), Columbia University (44; 7,207), the University of Cambridge (23; 6,816), and Stanford University (21; 6,431). Additional high-link-strength contributors include the University of Milan, Massachusetts General Hospital, the University of Sheffield, and Harvard Medical School (Supplementary Table 2; Supplementary Figures 2a,b).

### 3.4 Analysis of publishing authors

The author collaboration network reveals several tightly connected subgroups radiating from influential, high-productivity investigators (Supplementary Figure 3a). The time overlay shows that many of these authors initiated sustained research programs early in the period and remained active thereafter (Supplementary Figure 3b). By citations within this corpus, leading publishing authors include Beal M. Flint (21 documents; 7,365 citations), Beckman Joseph S. (20; 5,941), Shaw Christopher E. (11; 4,235), Cleveland Don W. (13; 3,600), Henderson Christopher E. (10; 2,704), Martin Lee J. (32; 2,607), Przedborski Serge (14; 2,603), Blair Ian P. (13; 2,429), Yuan Junying (12; 2,205), and Shaw Pamela J. (26; 2,202). Atkin Julie D., Ferraiuolo Laura, and Vargas Marcelo R. also occupy prominent positions with strong collaborative ties (Supplementary Table 3; Supplementary Figures 3a,b).

### 3.5 Analysis of publishing co-cited authors

Co-citation clustering delineates a stable knowledge backbone connecting landmark contributors across clusters (Supplementary Figure 4a). Temporal density underscores long-lasting influence of foundational work, with several authors remaining highly co-cited across many years (Supplementary Figure 4b). Top co-cited authors include Rosen D. R. (883 co-citations), Rothstein J. D. (858), Gurney M. E. (809), Martin L. J. (770), “Unknown” group entries associated with early foundational reports (704), Bruijn L. I. (661), Neumann M. (623), Pasinelli P. (542), Sasaki S. (465), and Wang J. (452). Beers D. R., Renton A. E., Mattson M. P., Beal M. F., and Yamanaka K. also figure prominently (Supplementary Table 4; Supplementary Figures 4a,b).

### 3.6 Analysis of publishing co-cited journals

**Figure 3:**
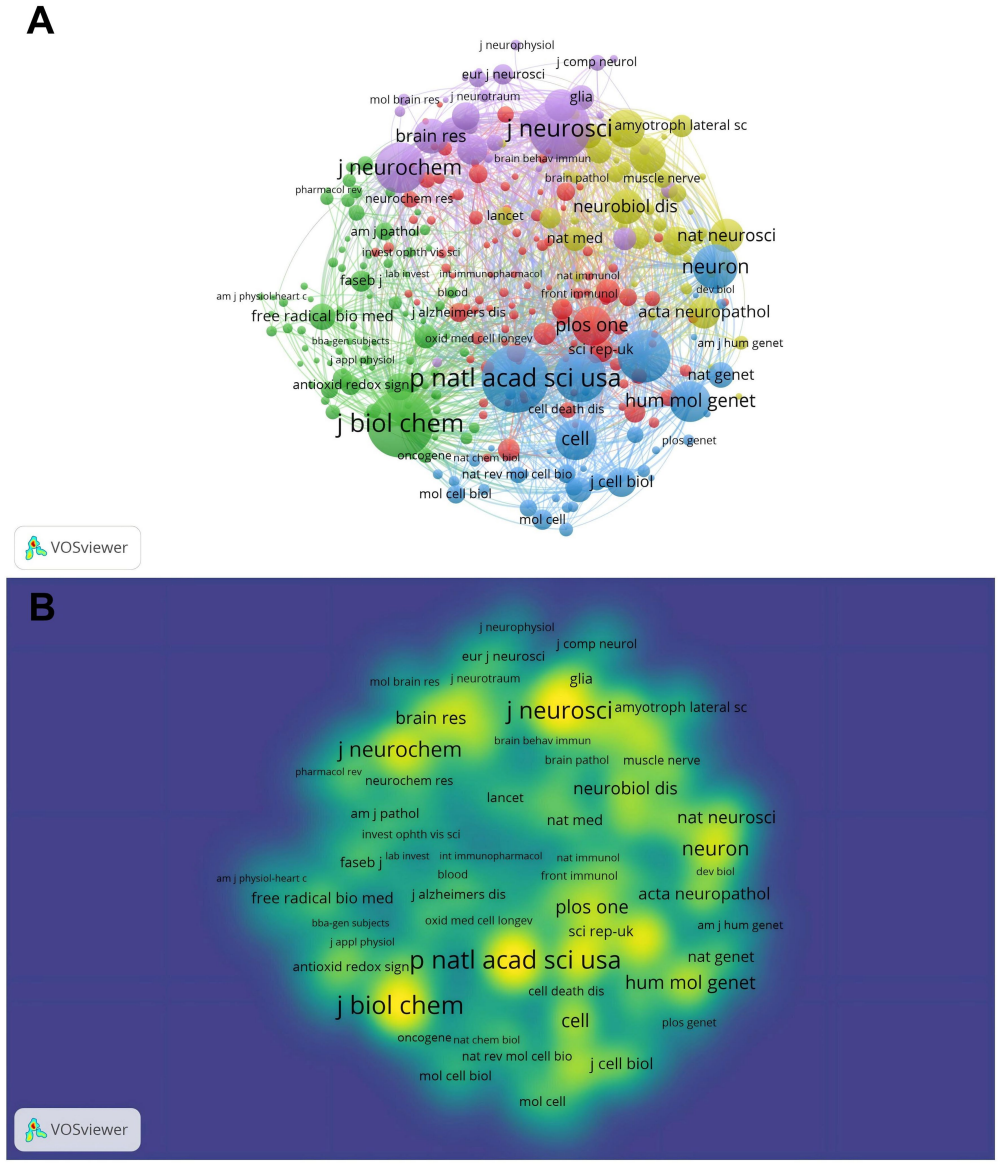
Cluster analysis of co-cited journals for publications (the minimum threshold for the node is 200): **(A)** Cluster analysis of co-cited journals for publications and **(B)** temporal heatmap of co-cited journals for publications.

The journal co-citation network is dominated by high-impact, cross-disciplinary titles in neuroscience and molecular biology, around which specialty journals aggregate (Figure 3A). The temporal heatmap shows persistently high attention for these outlets over the entire window (Figure 3B). By co-citations, Proceedings of the National Academy of Sciences (14,816) ranks first, followed by Journal of Biological Chemistry (14,516), Journal of Neuroscience (12,967), Science (9,760), Nature (9,566), Journal of Neurochemistry (9,121), Neuron (7,288), Human Molecular Genetics (6,264), Cell (6,176), and PLOS One (5,828). Annals of Neurology, Neurology, Brain Research, Neurobiology of Disease, Nature Neuroscience, Experimental Neurology, Acta Neuropathologica, Neuroscience Letters, and Journal of Cell Biology also serve as key co-citation hubs (Table 1; Figures 3A,B).

**Table 1:** Top 20 co-cited journals with the highest number of citations.

Source	Citations	Total link strength
P Natl Acad Sci USA	14,816	1,835,078
J Biol Chem	14,516	1,867,989
J Neurosci	12,967	1,611,297
Science	9,760	1,149,754
Nature	9,566	1,223,870
J Neurochem	9,121	1,199,020
Neuron	7,288	877,160
Hum Mol Genet	6,264	784,188
Cell	6,176	790,882
Plos One	5,828	694,755
Ann Neurol	5,594	678,622
Neurology	5,134	612,019
Brain Res	4,708	615,970
Neurobiol Dis	4,661	587,185
Nat Neurosci	4,448	520,143
Exp Neurol	4,192	505,695
Acta Neuropathol	4,002	463,446
Neurosci Lett	3,758	507,245
J Cell Biol	3,513	424,241
Brain	3,449	419,433

### 3.7 Analysis of publishing co-cited keywords

**Figure 4:**
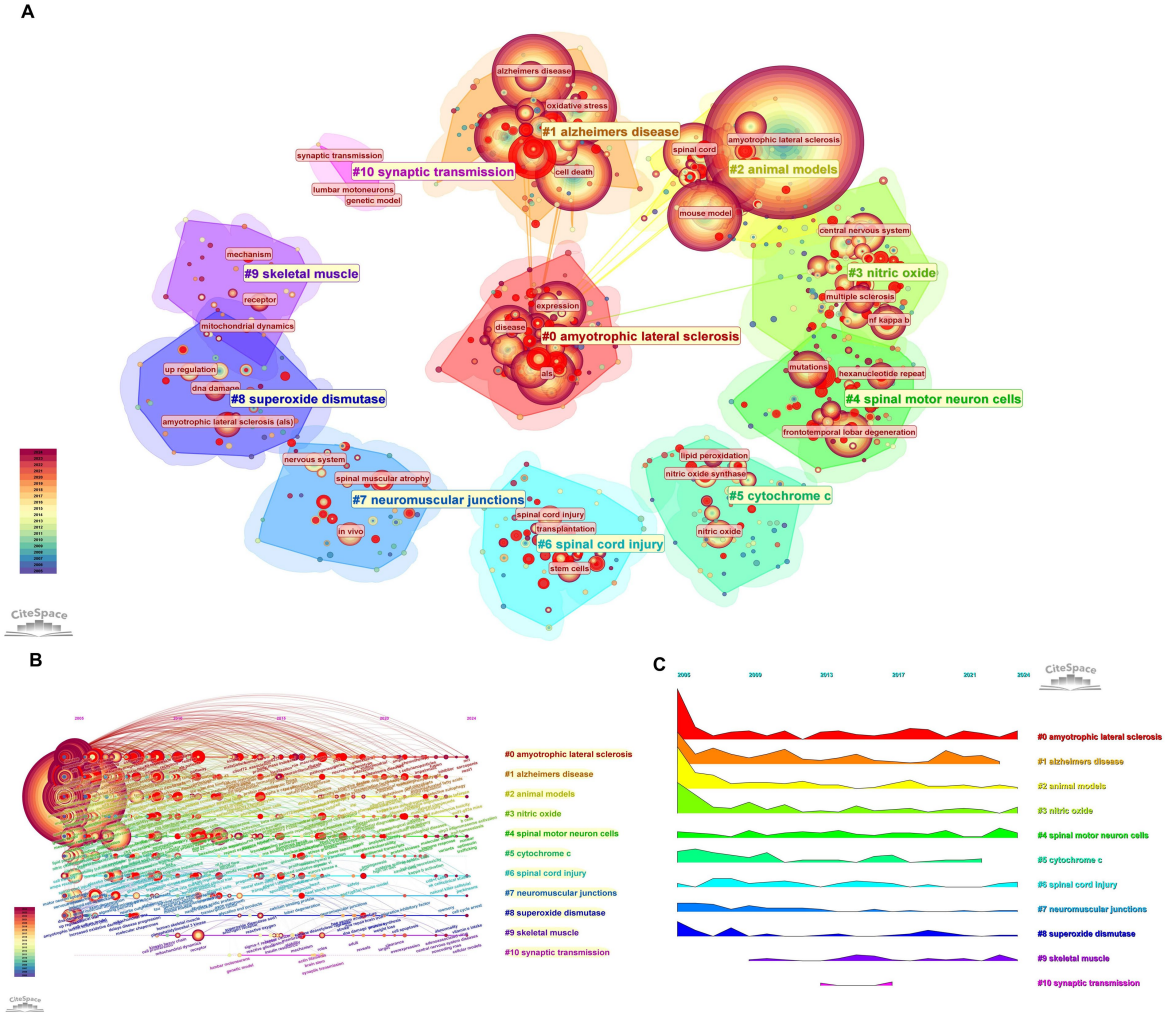
Keyword clustering analysis of published articles: Group 1 **(A)** keyword clustering analysis of published articles, **(B)** detailed display of keyword clustering of published articles, **(C)** trend chart of temporal burst changes of keywords in published articles.

**Figure 5:**
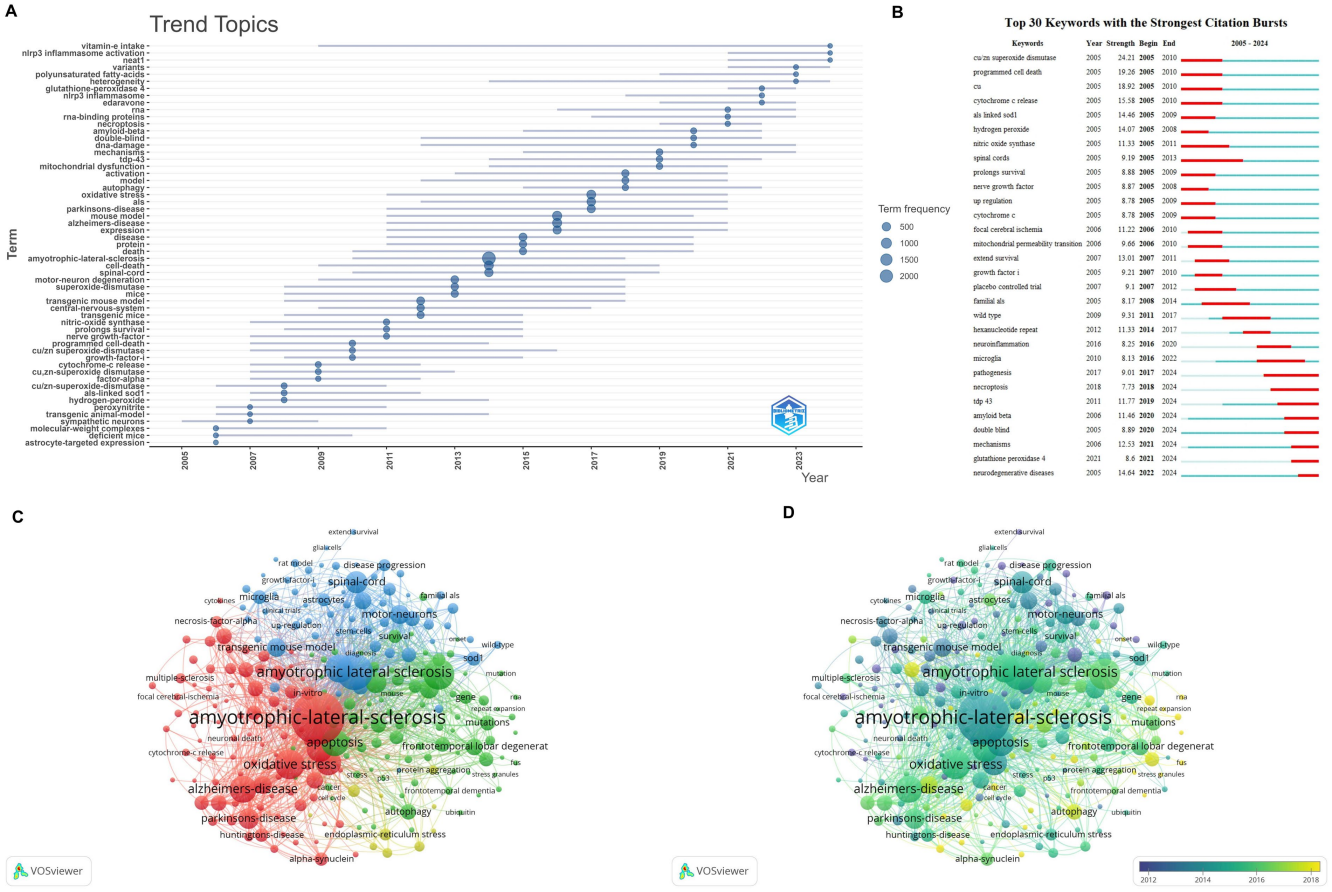
Cluster analysis and temporal dynamics of keywords in publications (minimum keyword occurrence = 30). **(A)** Timeline of topic clusters derived from keyword co-occurrence; each “topic” aggregates multiple related terms, and the displayed label denotes a representative/high-weight keyword within that cluster (not a manually written summary). **(B)** Top 30 burst keywords identified for individual terms based on abrupt increases in usage over time (burst intensity reflects temporal frequency change rather than thematic representativeness). **(C)** Keyword co-occurrence cluster map (VOSviewer). **(D)** Overlay/burst-time visualization of keyword dynamics (VOSviewer).

Keyword.

co-occurrence forms a layered thematic structure that spans disease entities ellular–molecular mechanisms models and translational foci. High-density cores are centered on “amyotrophic lateral sclerosis,” “oxidative stress,” “apoptosis,” “spinal cord,” and “motor neurons,” with tight intra-cluster linkages (Figures 4A–C). Trend and burst analyses show a thematic shift from classical oxidative stress and apoptosis toward multi-pathway regulated cell death and mitochondrial processes while research around transgenic mouse models and spinal pathways remains consistently active. Recent surges involve terms such as necroptosis mitophagy ferroptosis and neuroinflammation alongside enduring anchors like motor neuron transgenic mouse model and survival (Figures 5A–D)

To avoid confusion between panels that both appear “trend-like,” Figure 5A summarizes topic-level evolution by aggregating co-occurring keywords into clusters (a “topic” is shown as a cluster, and the displayed label is typically the highest-weight/most frequent representative term within that cluster). In contrast, Figure 5B reports keyword-level bursts for individual terms (i.e., sudden increases in usage frequency over time), which are not intended to be “representative” labels of a broader topic but rather indicators of temporal intensity; therefore, the two panels are complementary rather than expected to look identical.

### 3.8 Analysis of publishing co-cited literature

**Figure 6:**
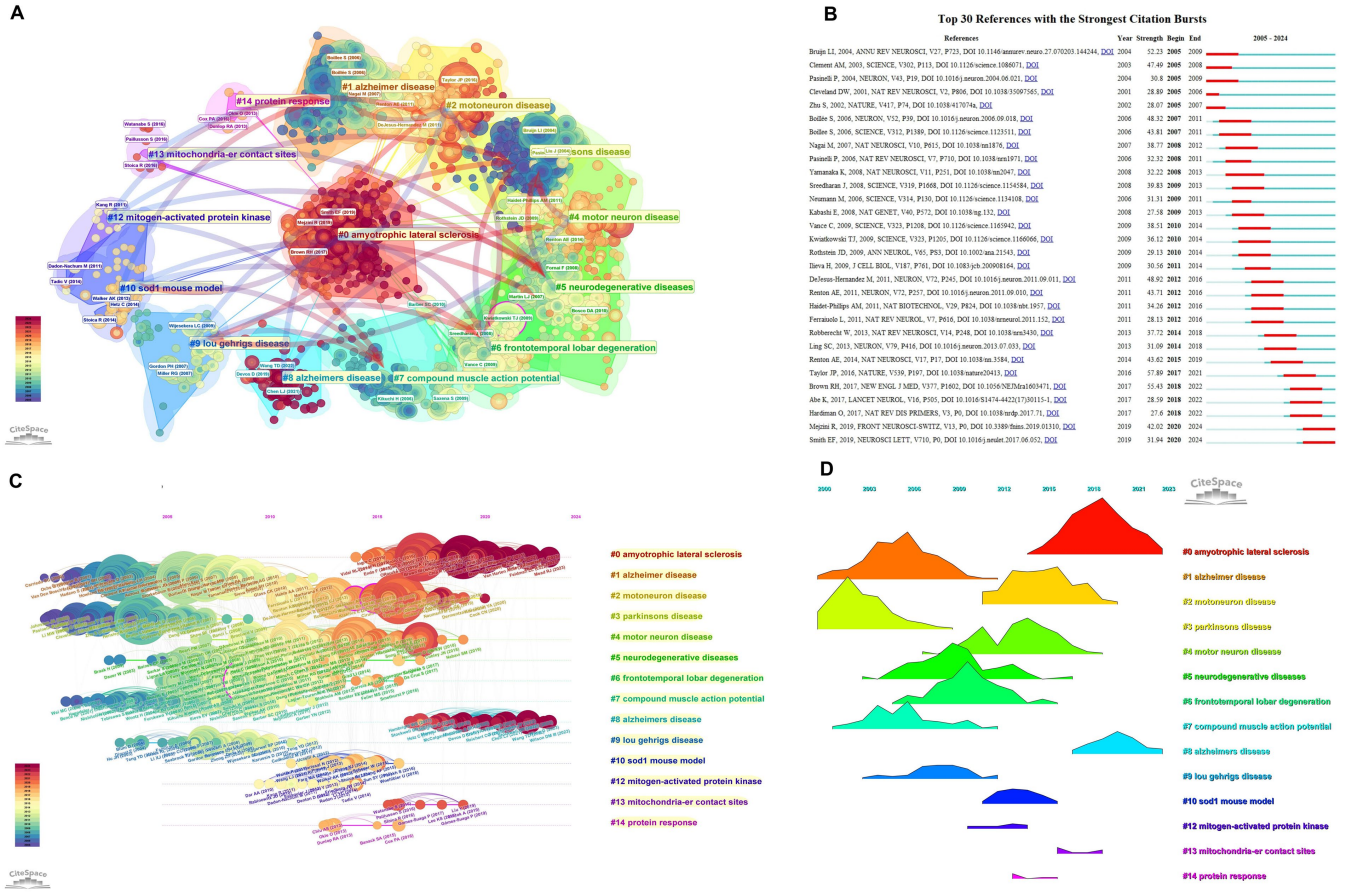
Analysis of co-cited publications: **(A)** Cluster analysis of co-cited publications, **(B)** burst years of the top thirty co-cited publications, **(C)** detailed cluster display of co-cited publications, and **(D)** temporal burst trend of co-cited publications.

Reference co-citation clusters chart the intellectual structure of the field, with major communities centered on “amyotrophic lateral sclerosis,” “Alzheimer disease,” “motor neuron disease,” “Parkinson’s disease,” and broader “neurodegenerative diseases” (Figure 6A). Burst detection highlights milestone studies that catalyzed shifts in attention at different times (Figure 6B). The temporal cluster landscape traces how foundational models and mechanisms evolved and cross-fertilized, identifying key nodes along each cluster’s trajectory (Figure 6C). Aggregate burst profiles indicate that the field progressed from model-driven discovery—such as SOD1 mouse work—toward integrated mechanism frameworks encompassing mitochondrial biology, protein homeostasis, frontotemporal lobar degeneration links, and motor-neuron pathology (Figure 6D).

### 3.9 Identification of shared gene signatures and functional pathways between ALS and regulated cell death

**Figure 7:**
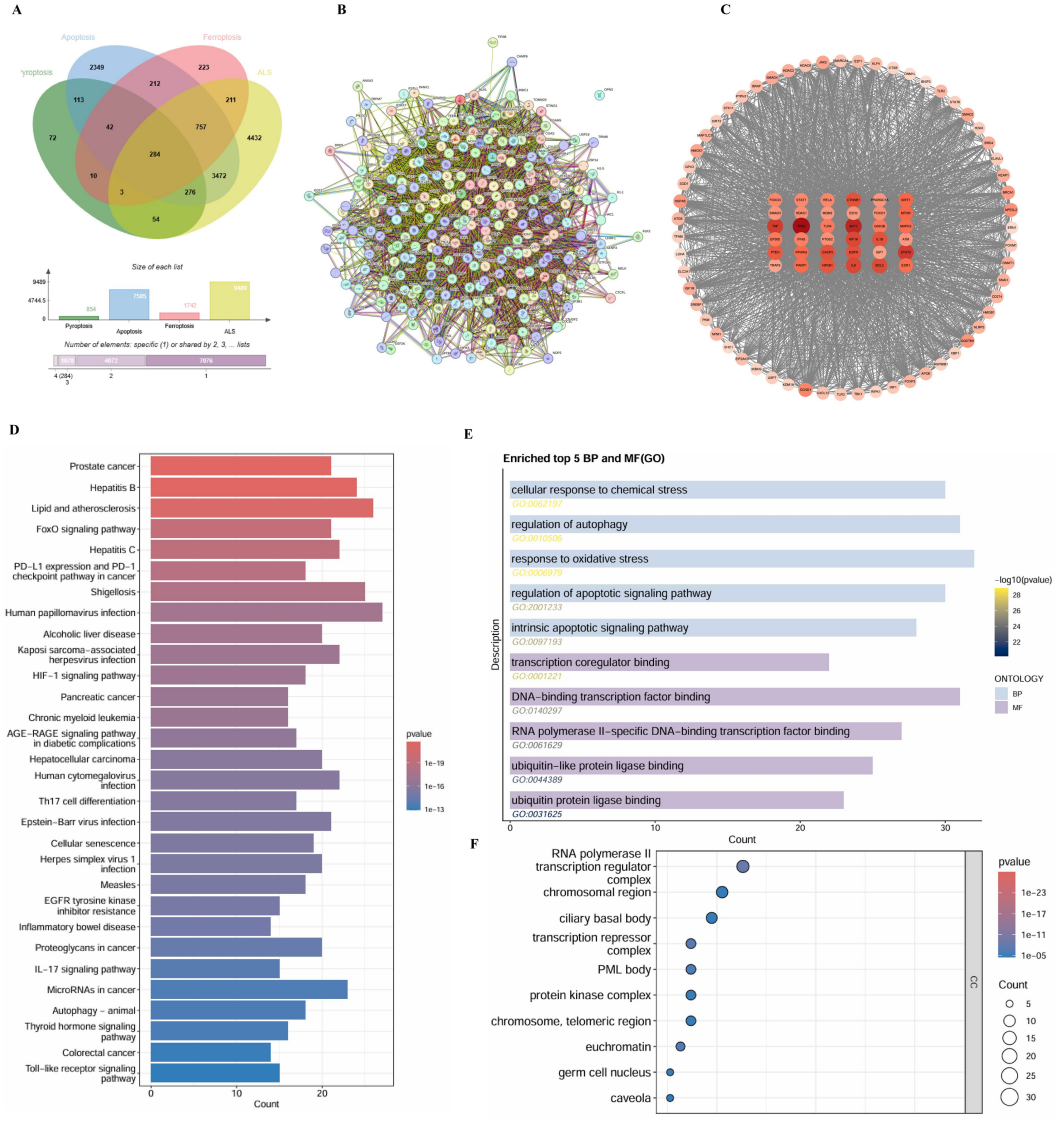
Landscape of shared gene signatures and functional enrichment analysis between ALS and regulated cell death. **(A)** Venn diagram illustrating the intersection of genes associated with ALS, apoptosis, ferroptosis, and pyroptosis. **(B)** Protein–protein interaction (PPI) network of the shared genes. **(C)** Visualization of the core interaction sub-network. **(D)** Bar chart showing the top enriched KEGG signaling pathways. **(E)** Bar chart displaying the top enriched Gene Ontology (GO) terms for biological processes (BP) and molecular functions (MF). **(F)** Dot plot representing the top enriched GO terms for cellular components (CC).

To uncover the potential molecular interplay between ALS and regulated cell death, we performed a comprehensive bioinformatics analysis. As illustrated in Figure 7A, the intersection of ALS-related genes with apoptosis, ferroptosis, and pyroptosis datasets revealed a significant number of shared targets, indicating a high degree of molecular convergence among these biological processes. Specifically, a substantial overlap was observed, suggesting that these cell death modalities share common genetic drivers with ALS. A protein–protein interaction (PPI) network was constructed for these shared genes to visualize their functional connectivity (Figure 7B), and a core interaction network was further extracted to highlight the most densely connected nodes (Figure 7C).

Pathway enrichment analysis provided further insights into the potential mechanisms. KEGG pathway analysis (Figure 7D) demonstrated that the shared targets were significantly enriched in pathways such as “Lipid and atherosclerosis,” “FoxO signaling pathway,” “HIF-1 signaling pathway,” and “PD-L1 expression and PD-1 checkpoint pathway in cancer,” as well as several infection-related pathways like “Hepatitis B,” “Human papillomavirus infection,” and “Shigellosis.” Gene Ontology (GO) analysis revealed that in terms of biological processes (BP) and molecular functions (MF) (Figure 7E), the targets were primarily involved in “cellular response to chemical stress,” “regulation of autophagy,” “response to oxidative stress,” and “regulation of apoptotic signaling pathway.” Binding activities such as “transcription coregulator binding” and “ubiquitin-like protein ligase binding” were also prominent. Regarding cellular components (CC) (Figure 7F), the targets were enriched in the “RNA polymerase II transcription regulator complex,” “chromosomal region,” “ciliary basal body,” and “transcription repressor complex.”

**Figure 8:**
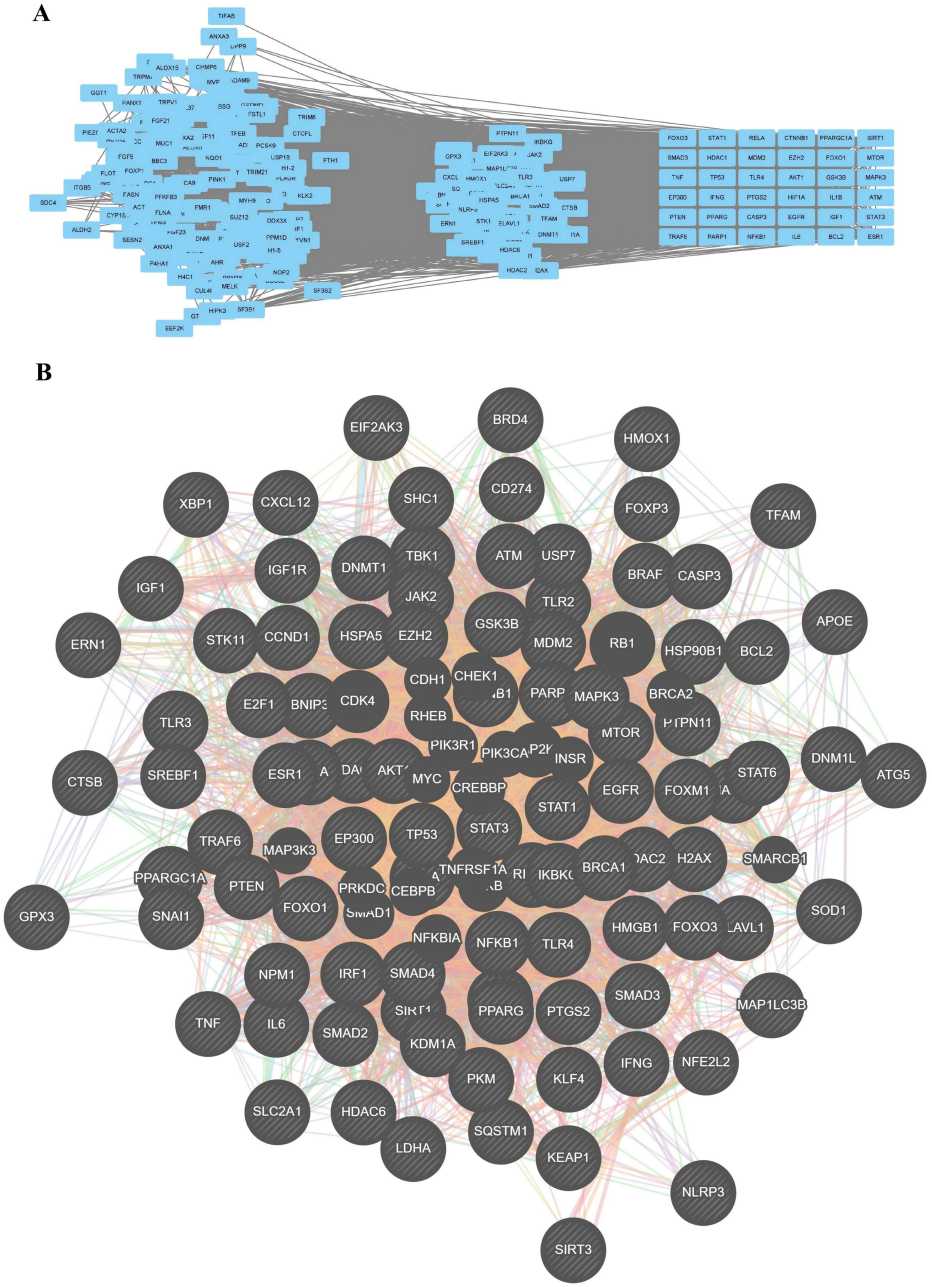
Identification of core protein interaction modules and hub genes. **(A)** Detection of densely connected gene clusters (modules) within the PPI network. **(B)** Network visualization of the top hub genes identified based on degree centrality, highlighting key regulatory nodes such as TP53, AKT1, STAT3, and MYC.

To further refine the key regulatory modules, we performed a clustering analysis, identifying three distinct gene modules with high internal connectivity (Figure 8A). From the global network, we identified the top hub genes based on degree centrality (Figure 8B). These core targets include TP53, AKT1, STAT3, MYC, EP300, CREBBP, RELA, HSP90AA1, JUN, and MAPK3. These hub genes likely serve as critical bridges connecting the pathogenic processes of ALS with ferroptosis, apoptosis, and pyroptosis.

## 4 Discussion

### 4.1 General information

Our bibliometric analysis of 6,272 records from 2005 to 2024 reveals a robust and accelerating global research effort at the interface of ALS and cell death, anchored by major contributions from the USA, China, and Italy. This expanding landscape reflects a deepening consensus that diverse cell death modalities are central to disease progression. Taken together, the map portrays a field that has matured around several, highly connected thematic poles—motor-neuron biology, mitochondrial signaling, oxidative/iron-dependent injury, and neuroinflammation—while progressively shifting its mechanistic center of gravity from “general neurodegeneration” toward named, regulated cell-death (RCD) programs. For the specific related search process (see Supplementary Material 2).

While national-level indicators show high overall productivity from China, Germany, and Japan, their individual institutions may appear less dominant in institutional rankings compared with top U.S. centers. Beyond listing representative institutions, this pattern likely reflects a structural dispersion of output: in these regions, ALS research and care are frequently organized through multi-center clinical networks and many affiliated hospitals/universities, which increases national totals but distributes publications and citations across numerous sites, thereby reducing the visibility of any single “mega-center” in institutional rankings (Atsuta and Sobue, 2010; Müller et al., 2018). In addition, bibliometric pipelines can fragment institutional credit due to address-reporting conventions, hospital–university dual affiliations, and multi-affiliation practices; such effects are well documented to influence institution-level indicators and rankings, especially when affiliation disambiguation systems differ (Donner et al., 2020; Hottenrott et al., 2017; Hottenrott et al., 2021). Consistent with this interpretation, important contributions from these countries are often distributed across multiple sites (e.g., multicenter registries/networks in Japan and Germany), even though leading hospitals still generate influential clinical cohorts and translational outputs—for example, longitudinal ALS clinic cohorts reported from major Chinese centers (Chen et al., 2023). Collectively, this “high national productivity–lower institutional concentration” pattern likely reflects decentralized, network-based research ecosystems rather than reduced research intensity, and it provides readers a useful structural lens for interpreting productivity maps and institutional rankings.

The prominence of clusters anchored in amyotrophic lateral sclerosis (ALS), animal models, synaptic transmission, nitric-oxide biology, cytochrome c, spinal motor-neuron cells, neuromuscular junctions (NMJ), superoxide dismutase (SOD1), and skeletal muscle suggests that investigators have converged on a shared causal chain: cortical and spinal network hyperexcitability increase glutamate drive and calcium flux; mitochondria integrate this stress with oxidative and iron signals; and motor neurons, muscle, and glia respond through specific death effectors whose signatures are now measurable in patients and models (Hardiman et al., 2017; Wang et al., 2022; Nógrádi et al., 2024).

Crucially, this bibliometric evolution is biologically substantiated by our bioinformatics findings, which identified a core network of hub genes—including TP53, AKT1, STAT3, and MYC—that serve as molecular bridges linking ALS pathogenesis with ferroptosis, apoptosis, and pyroptosis. For instance, TP53 (p53) has been recently established as a central regulator of motor neuron degeneration in C9orf72-ALS models, orchestrating a DNA-damage-induced death program distinct from classical apoptosis (Maor-Nof et al., 2021). Similarly, the enrichment of shared targets in the “FoxO signaling pathway” aligns with evidence that FoxO transcription factors are critical for regulating autophagy and proteostasis in motor neurons, a system often compromised in ALS (Du and Zheng, 2021). Furthermore, the identification of “Lipid and atherosclerosis” and “HIF-1 signaling” pathways echoes the growing appreciation of lipid peroxidation and metabolic dysregulation as central drivers of ferroptosis in motor neurons (Stockwell, 2022).

In parallel, citation “hotspots” in recent years increasingly reference ferroptosis, necroptosis, pyroptosis, and autophagy-lysosome dysfunction, echoing primary studies that place these pathways upstream of motor-neuron loss and, in some cases, directly link their pharmacologic modulation to disease modification (Wang et al., 2022; Ito et al., 2016; Zhang et al., 2022; Brenner et al., 2024). In our view, this bibliometric structure is not incidental—it mirrors a real, mechanistic consolidation in ALS, and it may explain why cell-death biology has become a nexus for both hypothesis generation and translational trials.

### 4.2 Common forms of cell death and ALS

#### 4.2.1 Neurodegeneration, ferroptosis, oxidative stress, and autophagy in ALS

Motor neuron degeneration in ALS emerges from a fragile metabolic equilibrium at the intersection of iron homeostasis, lipid metabolism, mitochondrial bioenergetics, and redox defense. Central to this vulnerability is ferroptosis, an iron-dependent, lipid-peroxidation–driven form of regulated cell death that is mechanistically distinct from apoptosis or necroptosis (Dixon et al., 2012).

Under physiological conditions, iron uptake via transferrin receptor (TFRC) and storage in ferritin (FTH1/FTL) tightly restrict the labile iron pool. However, in ALS, dysregulated ferritinophagy mediated by NCOA4 leads to excessive release of ferrous iron (Fe^2+^), which catalyzes Fenton reactions and accelerates lipid peroxidation in PUFA-enriched neuronal membranes (Mancias et al., 2014; Gao et al., 2016). In parallel, lipid-remodeling enzymes such as ACSL4 and LPCAT3 increase incorporation of polyunsaturated fatty acids into phospholipids, expanding the pool of substrates vulnerable to oxidative damage and defining the execution phase of ferroptosis (Doll et al., 2017; Kagan et al., 2017).

Mitochondrial dysfunction further amplifies this process. Defective electron transport chain activity promotes ROS spillover, which directly oxidizes membrane phospholipids and reinforces iron-driven lipid peroxidation (Lin and Beal, 2006; Cozzolino and Carrì, 2012). Normally, the System xc^−^–glutathione–GPX4 axis counteracts this threat by detoxifying lipid hydroperoxides. In ALS, however, excitotoxic inhibition of System xc^−^ reduces cystine uptake, limits glutathione synthesis, and compromises GPX4 activity, thereby weakening the principal antioxidant barrier against ferroptosis (Dixon et al., 2012; Stockwell et al., 2017).

**Figure 9:**
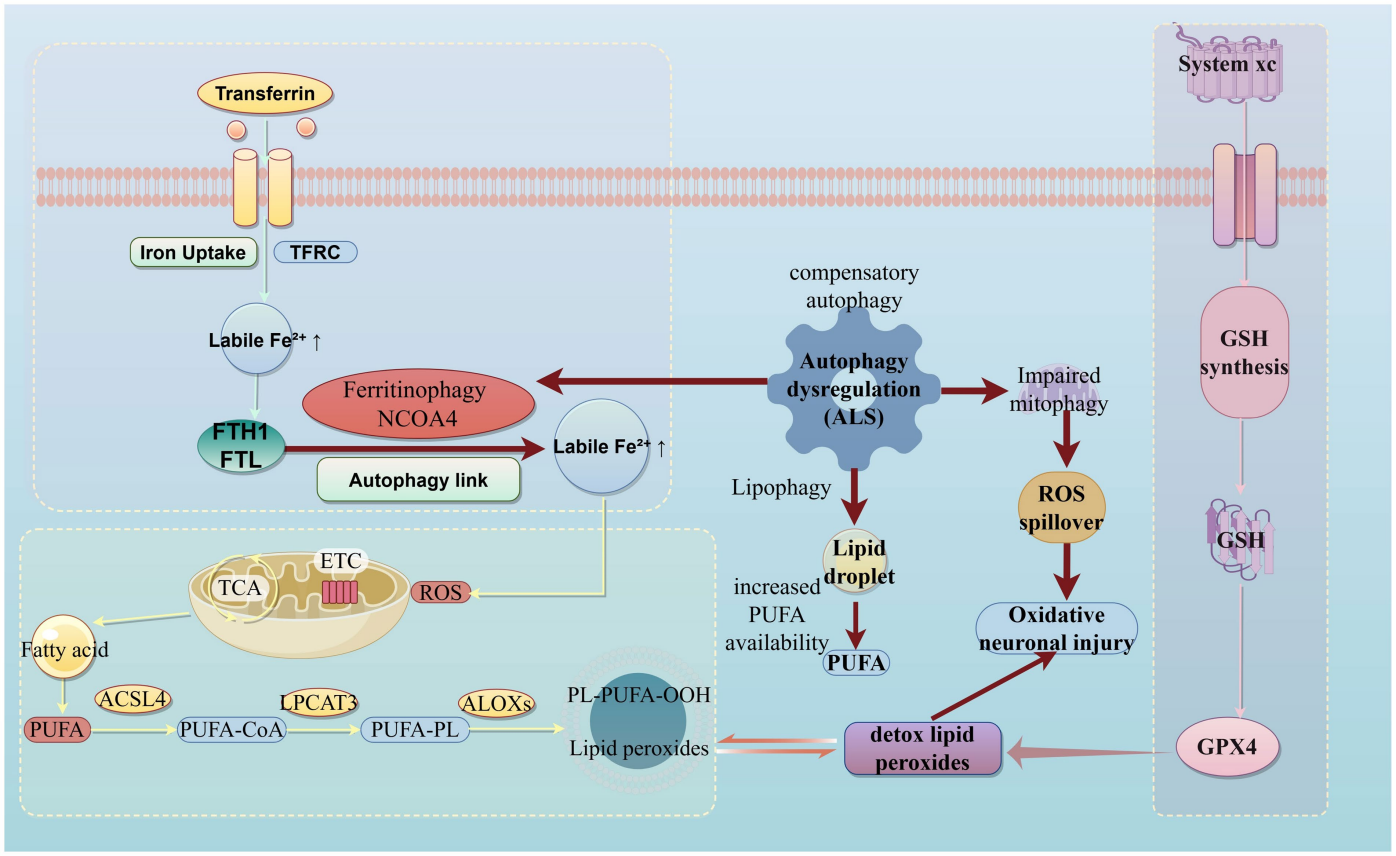
Metabolic convergence of ferroptosis, autophagy dysregulation, and antioxidant failure in ALS motor neuron degeneration (using figdraw). This schematic illustrates the integrated ferroptotic–autophagic–redox network underlying motor neuron vulnerability in amyotrophic lateral sclerosis (ALS). Upper left, iron-driven lipid peroxidation (ferroptosis axis): transferrin–TFRC–mediated iron uptake and NCOA4-dependent ferritinophagy increase the labile Fe^2+^ pool, facilitating Fenton chemistry and lipid peroxidation in PUFA-rich neuronal membranes. Lower left, PUFA–lipid peroxidation amplification module (ferroptosis execution phase): ACSL4 and LPCAT3 promote incorporation of polyunsaturated fatty acids (PUFAs) into membrane phospholipids, which are subsequently oxidized by lipoxygenases and mitochondrial ROS to generate toxic phospholipid hydroperoxides (PL-PUFA-OOH). Right, antioxidant and redox defense pathway (System xc^−^–GSH–GPX4): impaired cystine import via System xc^−^ limits glutathione (GSH) synthesis and compromises GPX4-mediated detoxification of lipid peroxides. Dysregulated autophagy in ALS links these modules by releasing excess iron (ferritinophagy), increasing PUFA availability (lipophagy), and failing to clear damaged mitochondria (impaired mitophagy), resulting in ROS spillover, oxidative neuronal injury, and progressive motor neuron degeneration.

Autophagy acts as a critical but double-edged modulator in this network. While basal autophagy and mitophagy are essential for neuronal homeostasis, selective autophagy pathways become maladaptive in ALS. Ferritinophagy releases additional iron, lipophagy increases PUFA availability, and impaired mitophagy allows damaged mitochondria to accumulate, collectively fueling oxidative stress and ferroptotic susceptibility (Gao et al., 2016; Masaldan et al., 2019). Thus, instead of protecting motor neurons, dysregulated autophagy amplifies the convergence of iron overload, lipid peroxidation, and mitochondrial ROS generation, culminating in progressive oxidative neuronal injury (Figure 9; Table 2).

**Table 2:** Plain-language summary of the three interconnected pathways illustrated in Figure 9.

Pathway	Key nodes shown in Figure 9	What this pathway normally does	What goes wrong in ALS	How it connects to the other pathways	Why this matters for motor neuron degeneration	Representative references
Iron-driven lipid peroxidation (ferroptosis axis)	Transferrin–TFRC, Ferritin (FTH1/FTL), Ferritinophagy (NCOA4), Labile Fe^2+^, PUFA, lipid peroxides	Maintains iron homeostasis by safely importing, storing, and using iron while minimizing oxidative damage to membranes	Dysregulated ferritinophagy releases excess reactive iron, increasing labile Fe^2+^ pools that catalyze lipid peroxidation	Excess iron amplifies mitochondrial ROS and accelerates oxidation of PUFA-rich membranes, directly feeding into oxidative stress pathways	Motor neurons contain iron-rich environments and PUFA-rich membranes, making them especially susceptible to iron-catalyzed lipid damage and ferroptotic injury	Dixon et al. (2012), Mancias et al. (2014), and Gao et al. (2016)
Oxidative stress and mitochondrial dysfunction	Mitochondria, ETC, ROS spillover	Generates cellular energy while keeping reactive oxygen species (ROS) at low, tightly controlled levels	Impaired mitochondrial quality control allows damaged mitochondria to accumulate and leak excessive ROS	Mitochondrial ROS further oxidize membrane lipids and overwhelm antioxidant systems, reinforcing ferroptosis and lipid peroxidation	Chronic oxidative stress damages neuronal membranes, proteins, and DNA, progressively impairing motor neuron function and survival	Lin and Beal (2006) and Cozzolino and Carrì (2012)
Autophagy dysregulation (ferritinophagy, lipophagy, impaired mitophagy)	Autophagy dysregulation (ALS), Ferritinophagy, Lipophagy, Impaired mitophagy	Removes damaged organelles and recycles iron, lipids, and proteins to preserve cellular homeostasis	Selective autophagy becomes maladaptive: excess iron and fatty acids are released, while damaged mitochondria are not efficiently cleared	Acts as a central modulator linking iron overload, increased PUFA availability, and mitochondrial ROS leakage	Instead of protecting neurons, dysregulated autophagy unintentionally fuels oxidative stress and ferroptotic vulnerability, accelerating motor neuron degeneration	Gao et al. (2016) and Masaldan et al. (2019)

This table summarizes the core biological functions, ALS-specific dysregulation, pathway interconnections, and pathogenic relevance of the three major mechanisms depicted in Figure 9, providing a simplified explanation for clinicians and non-specialist readers.

Furthermore, the relationship between autophagy and cell death in ALS appears to be highly context-dependent, transitioning from a survival mechanism to a lethal driver known as autophagy-dependent cell death. Figure 9 illustrates a compensatory autophagy response intended to recycle damaged organelles; however, under conditions of iron starvation or excessive oxidative pressure, this process can become maladaptive. Specific forms of selective autophagy, such as ferritinophagy and lipophagy, directly fuel the ferroptotic machinery. The nuclear receptor coactivator 4 (NCOA4) mediates the autophagic degradation of ferritin, liberating stored iron into the labile pool where it promotes free radical generation (Mancias et al., 2014; Gao et al., 2016). Simultaneously, excessive lipophagy releases free fatty acids that are rapidly esterified into membranes, providing fresh fuel for peroxidation. This “lethal autophagy” suggests that therapeutic strategies must carefully distinguish between restoring homeostatic flux and inhibiting these specific autophagic pathways that exacerbate metabolic collapse (Plaza-Zabala et al., 2017; Masaldan et al., 2019).

#### 4.2.2 Regulated necrosis in ALS

**Figure 10:**
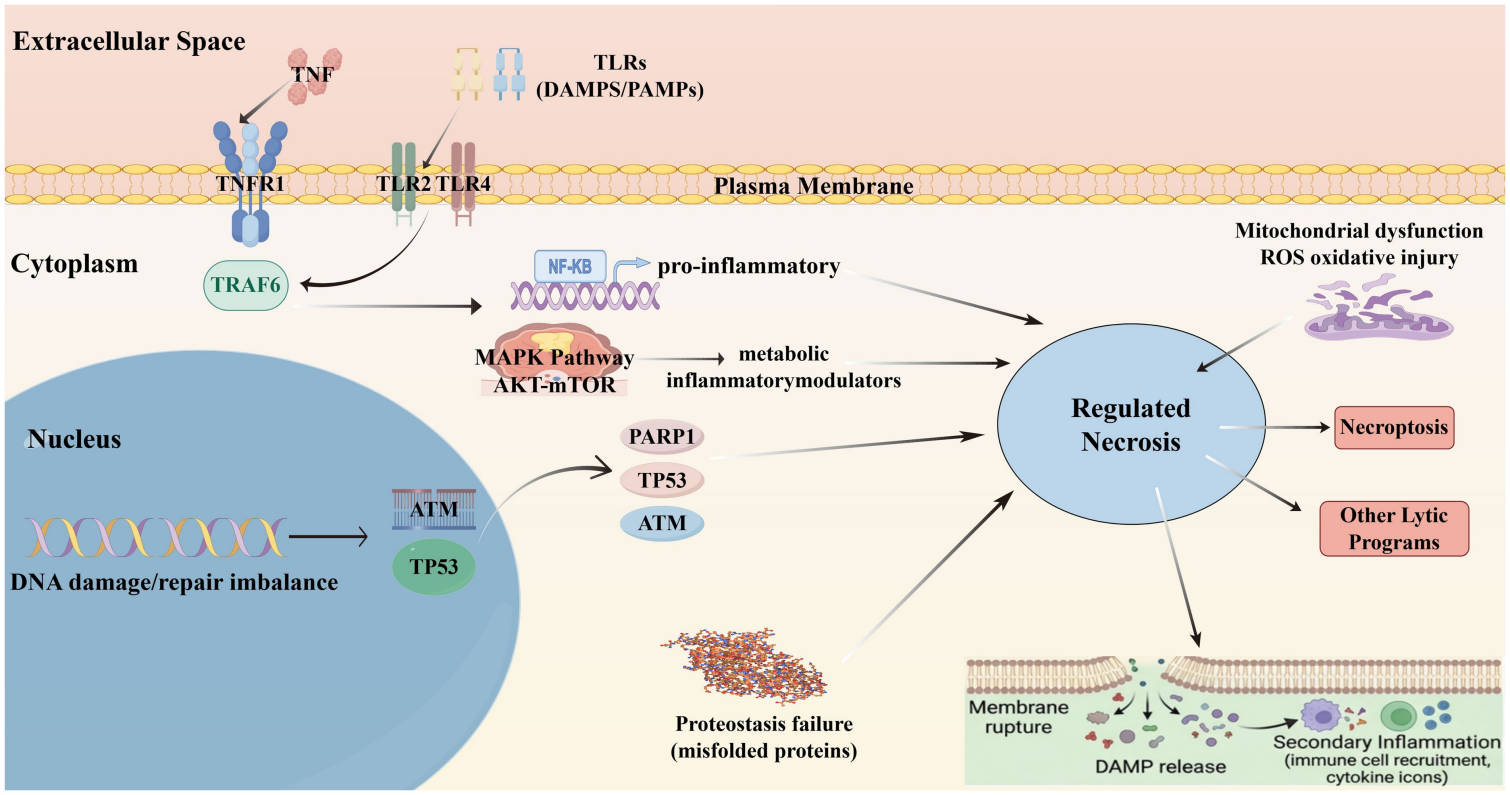
Signaling integration of inflammation, DNA damage, and regulated necrosis in the ALS microenvironment (using Figdraw).

Distinct from the metabolic collapse of ferroptosis, regulated necrosis in ALS represents an immunogenic mode of cell death orchestrated by a complex interplay between extracellular inflammatory signals and intracellular kinase networks. The pathway architecture depicted in Figure 10 highlights the critical role of the tumor necrosis factor (TNF) superfamily and pathogen/damage-associated molecular patterns (DAMPs) in initiating this process. Upon binding to TNFR1 or Toll-like receptors (TLRs), these ligands trigger the recruitment of adaptor proteins and kinases, including TRAF6, which occupies a central position in our protein–protein interaction network. In a healthy context, TRAF6 facilitates the ubiquitination required for NF-κB activation, promoting the transcription of pro-survival and pro-inflammatory genes (Hayden and Ghosh, 2014). However, in the chronic stress environment of ALS, the stability of this survival signaling is undermined. When caspase-8 activity is inhibited or the levels of RIPK1 and RIPK3 exceed a critical threshold, the receptor-interacting protein kinases assemble into the necrosome. This amyloid-like complex phosphorylates the pseudokinase MLKL, driving its oligomerization and translocation to the plasma membrane where it executes pore formation and membrane rupture (Pasparakis and Vandenabeele, 2015; Yuan et al., 2019).

The nuclear compartment also contributes significantly to this necrotic program through DNA damage response pathways. The accumulation of oxidative DNA lesions activates the sensor kinase ATM and the tumor suppressor p53, which we identified as top-ranking hubs in the ALS gene network. While this response initially attempts to arrest the cell cycle for repair, persistent genotoxic stress leads to the hyperactivation of PARP1. This enzyme consumes excessive amounts of NAD^+^ to synthesize poly (ADP-ribose) polymers, resulting in a bioenergetic crisis that depletes ATP pools required for apoptotic execution, thereby forcing the cell into a necrotic fate often termed parthanatos (Yang et al., 2024; Dawson and Dawson, 2017). This nuclear-mitochondrial crosstalk reinforces the necrotic trajectory, ensuring that cells with irreparable damage are swiftly eliminated (Figure 10).

Crucially, the execution of regulated necrosis transforms the dying motor neuron into a potent inflammatory stimulus, establishing a self-propagating cycle of tissue injury. The rupture of the plasma membrane releases intracellular DAMPs—such as HMGB1, ATP, and oxidized DNA—into the extracellular milieu. These molecules act as alarmins that sustain the activation of microglia and astrocytes, prompting them to secrete additional TNF, IL-1β, and neurotoxic factors (Kigerl et al., 2014; Liddelow et al., 2017). This secondary inflammation not only recruits peripheral immune cells but also lowers the death threshold for neighboring neurons, effectively spreading neurodegeneration in a “prion-like” manner. The integration of the inflammasome (e.g., NLRP3) in this process further amplifies the cytokine storm, suggesting that regulated necrosis is not merely an endpoint but a key propagation mechanism in ALS pathology (Ito et al., 2016; Ofengeim et al., 2017).

#### 4.2.3 ALS and pyroptosis

**Figure 11:**
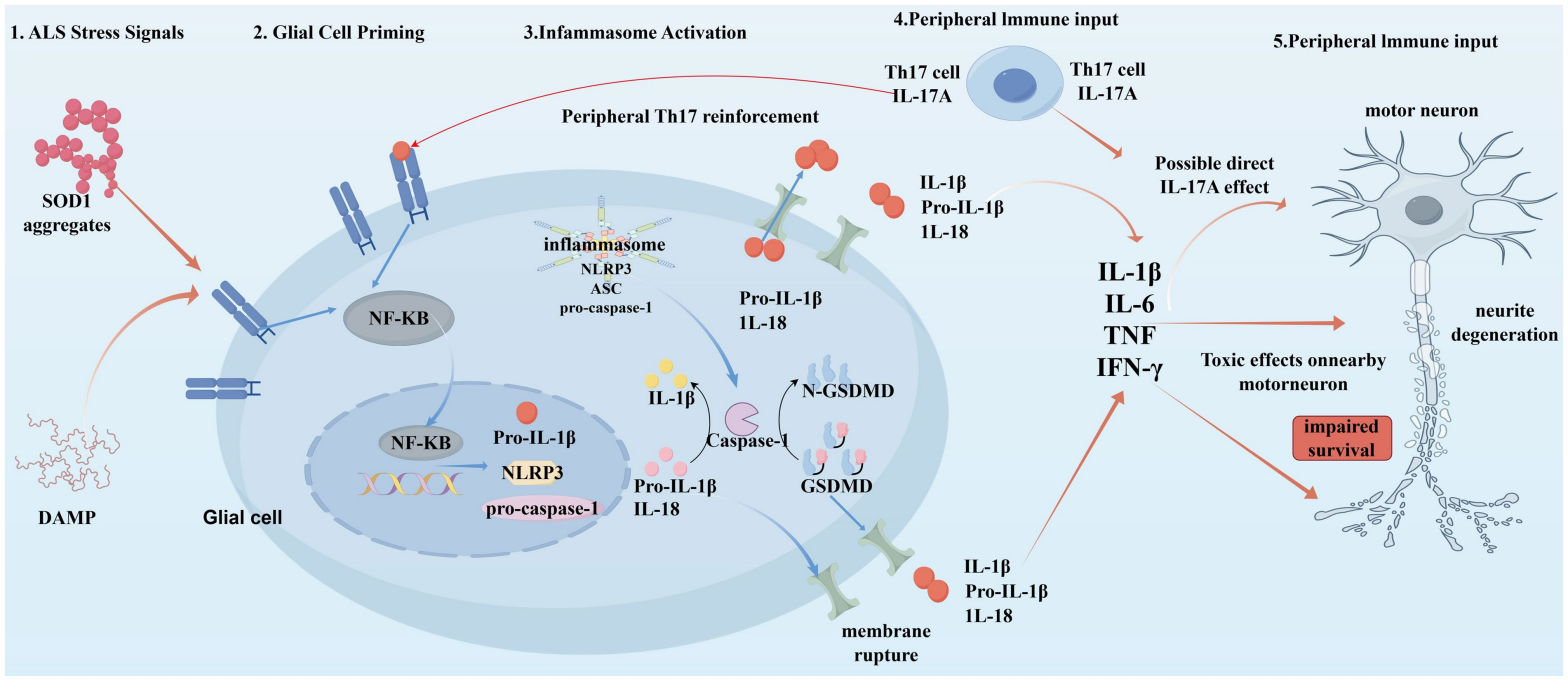
The feed-forward cycle of glial pyroptosis and peripheral immune infiltration in ALS pathogenesis (using figdraw). The schematic delineates the multistep activation of the NLRP3 inflammasome in glial cells and its downstream neurotoxic effects. (1) ALS stress signals: mutant SOD1 aggregates and DAMPs act as initial triggers. (2) Glial cell priming: activation of surface receptors leads to NF-κB nuclear translocation (signal 1), driving the transcription of NLRP3, pro-IL-1β, and pro-caspase-1. (3) Inflammasome activation: upon secondary stimulation, the NLRP3-ASC-Caspase-1 complex assembles, leading to Caspase-1 activation. This results in the maturation of IL-1β/IL-18 and the cleavage of Gasdermin D (GSDMD), causing membrane rupture (pyroptosis) and cytokine release. (4) Peripheral immune input: infiltrating Th17 cells secrete IL-17A, which creates a positive feedback loop reinforcing glial activation and potentially exerting direct toxicity. (5) Neurotoxicity: the resulting cytokine storm (IL-1β, TNF, IL-6, IFN-*γ*) targets adjacent motor neurons, causing neurite degeneration and impaired survival.

The trajectory of neuroinflammation in ALS is fundamentally driven by pyroptosis, a lytic and highly immunogenic form of regulated cell death that predominates in the glial compartment. As delineated in our proposed mechanism (Figure 11), this cascade initiates when glial pattern recognition receptors (PRRs) detect ALS-specific stress signals, specifically mutant SOD1 aggregates and damage-associated molecular patterns (DAMPs) released from dying neurons. This engagement triggers the canonical NF-κB signaling pathway—anchored by the RELA hub identified in our network analysis—which serves as the “priming” signal (Signal 1). This transcriptional phase is critical for upregulating the expression of rate-limiting inflammasome components, including the sensor NLRP3, the adaptor ASC, and the zymogen pro-caspase-1, thereby establishing a molecular platform ready for activation (Deora et al., 2020; Bellezza et al., 2018).

Following this priming step, a secondary stimulus induces the assembly of the NLRP3 inflammasome complex, catalyzing the autocleavage and activation of caspase-1. This executioner protease performs two distinct but synergistic functions: it processes the pro-inflammatory cytokines IL-1β and IL-18 into their biologically active forms, and simultaneously cleaves Gasdermin D (GSDMD) to release its N-terminal pore-forming domain. The insertion of GSDMD-N into the plasma membrane creates large oligomeric pores that disrupt cellular osmotic balance, leading to membrane rupture and the explosive release of cytoplasmic contents (Broz and Dixit, 2016; Johann et al., 2015). This lytic event not only expels the processed cytokines but also releases additional alarmins, thereby converting a cell-intrinsic stress response into a propagative inflammatory wave that compromises the local microenvironment.

Crucially, this glial pyroptosis does not occur in isolation but is reinforced by a systemic immune dysregulation involving peripheral T-cell infiltration. As illustrated in the model, Th17 cells migrate across the compromised blood–brain barrier and secrete Interleukin-17A (IL-17A), which acts as a potent feedback accelerator on glial receptors to sustain the NF-κB-driven inflammatory state. This peripheral-central crosstalk amplifies the cytokine storm—comprising IL-1β, IL-6, TNF, and IFN-*γ*—which acts directly on motor neurons to induce neurite degeneration and trigger apoptotic or necroptotic death (Fiala et al., 2010; Rentzos et al., 2010). The elevation of circulating Th17 markers and the imbalance between Th17 and regulatory T cells (Tregs) observed in ALS patients further corroborate this pathogenic axis, suggesting that pyroptosis serves as a key node linking systemic autoimmunity with localized motor neuron degeneration (Saresella et al., 2013; Jin et al., 2020; Johann et al., 2015).

### 4.3 Immunometabolic interfaces and clinical heterogeneity

The translation of cellular death mechanisms into clinical phenotypes is heavily modulated by the systemic immune response, particularly the adaptive arm involving T-lymphocytes. While our network analysis highlights the primacy of glial-driven pyroptosis, emerging evidence suggests that the trajectory of disease progression is dictated by the peripheral balance between neuroprotective regulatory T cells (Tregs) and neurotoxic T helper 17 (Th17) cells. Tregs normally suppress the hyperactive microglial states that drive ferroptosis and necroptosis; however, in rapidly progressing ALS patients, the circulating Treg population is often functionally impaired and reduced in number (Henkel et al., 2013; Beers and Appel, 2019). This immunological dichotomy provides a biological basis for prognostic heterogeneity. Notably, this phenomenon is not unique to ALS; recent investigations into Alzheimer’s disease have similarly established that T-lymphocyte proportions serve as critical prognostic indicators, reinforcing the concept that peripheral immune tracking can predict neurodegenerative trajectories across different pathologies (Willman et al., 2024). In ALS, an expanded Th17 population secretes proinflammatory cytokines that compromise the blood-spinal cord barrier and reinforce lytic death pathways. Therefore, therapeutic strategies must extend beyond the CNS to restore systemic immune homeostasis, potentially utilizing autologous Treg infusions to arrest the feed-forward inflammatory cycle (Thonhoff et al., 2018).

Furthermore, this immunometabolic dysregulation extends beyond motor symptoms to influence the broader behavioral and cognitive profile of the disease. A significant proportion of ALS patients manifest deficits within the frontotemporal dementia (FTD) spectrum, characterized by apathy, executive dysfunction, and loss of empathy (Strong et al., 2017). Recent neuroimaging and fluid biomarker studies indicate that patients with pronounced behavioral impairment exhibit widespread cortical hyperexcitability and elevated markers of lipid peroxidation, mirroring the ferroptotic signatures identified in our clusters (Higashihara et al., 2021). Recognizing these behavioral changes as direct clinical readouts of the underlying cell death pathology is crucial. As demonstrated in broader neuropsychiatric contexts, granular behavioral analysis—ranging from symptom tracking to therapeutic response assessment—provides a sensitive metric for evaluating disease progression that complements traditional motor scoring (Lucke-Wold et al., 2025; Crockford et al., 2018). We posit that such detailed behavioral phenotyping in ALS could reveal subtle fluctuations in neuroinflammation before irreversible motor loss occurs (Lillo et al., 2010).

### 4.4 Emerging horizons: cross-disease mechanisms and AI-driven discovery

The molecular landscapes charted in this study reveal profound mechanistic convergences between ALS and other neurodegenerative conditions. Our co-citation clusters indicate that ferroptosis and mitophagy defects are not unique to ALS but represent “pan-neurodegenerative” failures of cellular quality control. For instance, the iron-dependent accumulation of lipid ROS is a central feature of dopaminergic neuron loss in Parkinson’s disease, while the inhibition of necroptosis has shown neuroprotective effects in Alzheimer’s models (Guiney et al., 2017; Reichert et al., 2020). These parallels suggest that ALS research can leverage mechanistic insights established in adjacent fields. Specifically, the concept of a “threshold of lethality”—where chronic proteotoxic stress synergizes with acute mitochondrial failure to trigger regulated cell death—appears to be a universal principle. Consequently, drugs developed to block ferroptosis or boost mitophagy in other proteinopathies could be rapidly repurposed for ALS, provided they target the specific lipid metabolism of the motor system (Ashraf et al., 2020).

Finally, the exponential growth of multi-omics data necessitates the integration of artificial intelligence (AI) and machine learning (ML) to decipher the complex regulatory networks governing cell death. Traditional reductionist approaches often fail to capture the non-linear interactions between the hundreds of genes identified in our PPI networks. Emerging AI-driven frameworks are now capable of integrating transcriptomic, proteomic, and clinical data to construct predictive models of disease progression and identify non-obvious therapeutic targets (Grollemund et al., 2019). These computational tools can distinguish between patient subgroups driven primarily by inflammatory necrosis versus those driven by metabolic ferroptosis, enabling a precision medicine approach (Sharifi-Rad et al., 2022). By applying deep learning algorithms to the vast bibliometric and biological datasets analyzed here, future research can move from static pathway maps to dynamic, patient-specific models of neurodegeneration, ultimately accelerating the discovery of biomarkers that signal the earliest phases of cellular distress.

### 4.5 Cell death in other neurodegenerative diseases

Regulated cell death (RCD) programs implicated in ALS also operate broadly across neurodegenerative disorders, where disease-specific proteinopathies and cell-type vulnerabilities converge on a partially shared set of execution pathways. Alzheimer’s disease (AD), Parkinson’s disease (PD), Huntington’s disease (HD), and frontotemporal dementia (FTD) all feature chronic proteotoxic stress, mitochondrial dysfunction, iron/redox imbalance, and maladaptive neuroinflammation—conditions that can lower the threshold for ferroptosis, necroptosis, pyroptosis, and autophagy–lysosome pathway failure (Kampmann, 2024; Gadhave et al., 2024). Importantly, these mechanisms are not interchangeable “labels,” but reflect distinct biochemical routes to neuronal injury that may dominate in different anatomical regions, stages, or cellular compartments. Table 3 provides a plain-language, cross-disease mapping of dominant triggers, representative RCD modules, and key molecular nodes, highlighting where mechanistic insights from adjacent fields can inform ALS-targeted hypothesis building and therapeutic prioritization.

**Table 3:** Plain-language summary of dominant regulated cell death (RCD) mechanisms across major neurodegenerative diseases and their mechanistic links to ALS.

Disease	Core pathological triggers	Dominant/reported RCD programs	Representative molecular nodes	Mechanistic relevance to ALS	Representative references
AD	Aβ plaques; tau pathology; chronic microglial activation	Pyroptosis/inflammasome-linked inflammatory injury; necroptosis-like inflammatory necrosis; iron/redox-linked lipid peroxidation; ALP dysfunction	NLRP3–caspase-1; ASC specks; RIPK1 axis; oxidative stress nodes; autophagy–lysosome impairment	Supports the concept that inflammasome priming + proteotoxic stress can amplify lytic/inflammatory neuronal injury and propagate pathology; parallels ALS neuroinflammatory amplification	Heneka et al. (2013), Venegas et al. (2017), and Yuan et al. (2019)
PD	α-synuclein pathology; iron accumulation; mitochondrial dysfunction in dopaminergic neurons	Ferroptosis susceptibility (iron-dependent lipid peroxidation); inflammatory necrosis amplification under chronic stress	Iron–lipid–ROS coupling; lipid peroxidation control points; mitochondrial ROS; innate immune stress programs	Reinforces iron–lipid–ROS as a cross-disease axis; offers rationale for ferroptosis-oriented therapeutic hypotheses relevant to ALS	Guiney et al. (2017) and Do Van et al. (2016)
HD	Mutant huntingtin (polyQ); proteostasis overload; impaired cargo handling	Autophagy–lysosome pathway failure (upstream); secondary oxidative stress and downstream RCD permissiveness	Beclin-1–dependent autophagy; impaired autophagic flux; mitochondrial damage accumulation	Provides a model for how ALP failure can act upstream, lowering resilience and enabling multiple downstream RCD endpoints	Menzies et al. (2017) and Nixon (2013)
FTD	TDP-43/tau proteinopathies; ALS–FTD spectrum genetics	Autophagy/quality-control failure as a primary vulnerability; downstream inflammatory amplification and neuronal death	Autophagy induction affecting TDP-43 turnover; TBK1-linked selective autophagy signaling	Highlights that clearance pathway defects (autophagy/selective QC) can be causal and trans-diagnostic across ALS–FTD	Barmada et al. (2014) and Freischmidt et al. (2015)

This table highlights core pathological triggers, representative RCD programs, and key molecular nodes reported in Alzheimer’s disease (AD), Parkinson’s disease (PD), Huntington’s disease (HD), and frontotemporal dementia (FTD). The final column provides representative references in cross-citation format to facilitate quick verification and to guide readers toward mechanistic entry points relevant to ALS cross-disease learning.

#### 4.5.1 Cell death mechanisms in Alzheimer’s disease (AD)

AD is characterized by extracellular Aβ deposition and intracellular tau pathology, accompanied by microglial activation and sustained neuroinflammation. Inflammasome signaling is particularly well supported in AD: NLRP3–caspase-1 activation has been demonstrated in AD brains and in APP/PS1 models, where genetic disruption of inflammasome components mitigates pathology (Heneka et al., 2013). Mechanistically, inflammasome activation can promote pyroptosis-like inflammatory injury through caspase-1–dependent cytokine maturation and gasdermin-mediated membrane rupture, amplifying glial–neuronal crosstalk (Wu et al., 2023). Beyond cytokines, ASC “specks” released from microglia can cross-seed Aβ, providing a plausible route for feed-forward propagation of proteinopathy alongside inflammatory damage (Venegas et al., 2017). In parallel, inflammatory necrosis programs and iron-driven lipid peroxidation have both been linked to AD-relevant stress contexts, consistent with the broader concept that chronic protein aggregation plus metabolic/redox stress can converge on lytic or ferroptotic endpoints (Zhang et al., 2017; Yuan et al., 2019). Autophagy–lysosome dysfunction further exacerbates this convergence by impairing clearance of Aβ/tau species and damaged organelles, thereby reinforcing oxidative stress and inflammatory signaling (Nixon, 2013).

#### 4.5.2 Cell death mechanisms in Parkinson’s disease (PD)

PD is defined by selective degeneration of dopaminergic neurons and *α*-synuclein pathology, in a milieu notable for iron accumulation, mitochondrial impairment, and oxidative stress. These features align closely with ferroptosis biology, and ferroptosis has been proposed as a mechanistically relevant contributor to dopaminergic neuron loss, where iron-dependent lipid peroxidation and antioxidant insufficiency can become dominant stress amplifiers (Guiney et al., 2017). Experimental work has further supported ferroptosis-like death in PD models and highlighted regulatory control points, strengthening the rationale for targeting ferroptotic susceptibility in dopaminergic systems (Do Van et al., 2016). Inflammatory RCD programs may also contribute in PD through sustained innate immune activation and mitochondrial danger signaling, creating conditions permissive for necroptotic/pyroptotic amplification, particularly in later disease stages (Yuan et al., 2019). Collectively, PD reinforces the concept that iron–lipid–ROS coupling is a cross-disease axis that may be especially informative for ALS, given the parallel appearance of ferroptosis-linked signals in ALS motor neuron vulnerability.

#### 4.5.3 Cell death mechanisms in Huntington’s disease (HD)

HD is caused by polyglutamine-expanded mutant huntingtin, producing profound proteostasis stress and neuronal dysfunction with prominent defects in cargo recognition and autophagic flux. Autophagy impairment is not merely secondary in HD; polyglutamine context can directly regulate Beclin-1–dependent autophagy and thereby influence aggregate handling and neuronal survival (Menzies et al., 2017). Autophagy–lysosome dysfunction also promotes mitochondrial damage accumulation, elevating ROS and increasing the likelihood that neurons cross a lethality threshold into regulated death programs under chronic stress (Nixon, 2013). In this sense, HD provides a well-studied framework for how impaired quality control (autophagy/lysosome) can function upstream of multiple RCD endpoints, including ferroptosis- and necrosis-like injury, by progressively constraining cellular buffering capacity.

#### 4.5.4 Cell death mechanisms in frontotemporal dementia (FTD)

FTD encompasses heterogeneous proteinopathies (notably TDP-43 and tau subtypes) and shares a genetic/clinical continuum with ALS. Many ALS/FTD-linked factors participate in selective autophagy and mitochondrial quality control, making autophagy–lysosome failure a core vulnerability that can indirectly potentiate downstream RCD programs. For example, enhancing autophagy can accelerate TDP-43 turnover and improve neuronal survival in ALS-relevant neuronal models, underscoring the causal linkage between proteostasis/autophagy control and neurotoxicity (Barmada et al., 2014). In addition, haploinsufficiency of TBK1—an ALS/FTD gene with key roles in selective autophagy and inflammatory signaling—provides genetic evidence that impaired quality-control signaling can predispose to neurodegeneration across the ALS–FTD spectrum (Freischmidt et al., 2015). Therefore, FTD most clearly illustrates how upstream “clearance pathway” defects can unify proteinopathy spread, mitochondrial stress, and inflammatory amplification, creating a permissive landscape for multiple execution modes of neuronal loss.

## 5 Conclusion

This study integrates a comprehensive bibliometric analysis of global research trends with a systems-level bioinformatics reconstruction to demonstrate that the conceptual framework of ALS pathogenesis has fundamentally shifted from generic neurodegeneration toward specific, actionable regulated cell death modalities. The convergence of citation hotspots and shared gene signatures—anchored by critical hubs such as TP53, AKT1, and RELA—identifies ferroptosis and pyroptosis as the dominant executioner pathways driving motor neuron loss. Our mechanistic mapping reveals that this lethality arises from a synergistic failure of mitochondrial bioenergetics and antioxidant defenses, which renders neuronal membranes susceptible to catastrophic lipid peroxidation while simultaneously triggering a glial-mediated inflammatory feedback loop involving the NLRP3 inflammasome and gasdermin D pore formation. These findings suggest that the clinical heterogeneity of ALS likely reflects the variable engagement of these interconnected metabolic and immune modules, fueled by systemic T-lymphocyte imbalances and maladaptive autophagy. Consequently, the field must move beyond reductionist approaches toward multimodal therapeutic strategies that simultaneously restore mitochondrial quality control, inhibit ferroptotic cascades, and modulate neuroinflammation, utilizing emerging artificial intelligence tools to identify patient-specific biomarkers for early intervention.

## 6 Limitations

This study previously acknowledged the use of a single database; after the update we searched three sources—Web of Science Core Collection (WoSCC), PubMed, and Scopus—so the one-database constraint has been resolved.

Several limitations remain. First, we restricted the search to English-language articles and reviews and to the time window 2005–2024. Because only seven records were available as of 08 November 2025, we excluded 2025 from the trend analyses; this choice may attenuate very recent changes. Second, differences in indexing practices and metadata across WoSCC, PubMed and Scopus can introduce residual selection or de-duplication bias, despite harmonization. Third, keyword-based strategies may have missed records that used atypical terminology for cell-death pathways or ALS. Fourth, citation-based indicators are time-dependent and sensitive to field size, so influence rankings should be interpreted cautiously. At the same time, Country / Region and institution attributions were derived from author affiliations at the time of publication and may not fully reflect the contribution structure of multi-institution collaborations.

Finally, although we applied predefined tool-specific priorities for different indicators, minor rank-order variations may still arise from software-specific implementations. These variations did not affect the overall patterns or conclusions of the study.

## Data Availability

The original contributions presented in the study are included in the article/Supplementary material, further inquiries can be directed to the corresponding authors.
