# Supplementary material for: From knowledge landscapes to network mechanisms: charting regulated cell death pathways in ALS
Source: Front Aging Neurosci. 2026 Jan 30;18:1742805. doi: 10.3389/fnagi.2026.1742805 (PMC12901334; doi:10.3389/fnagi.2026.1742805)
Supplement: Supplementary file 1 [file Data_Sheet_1.docx]

| 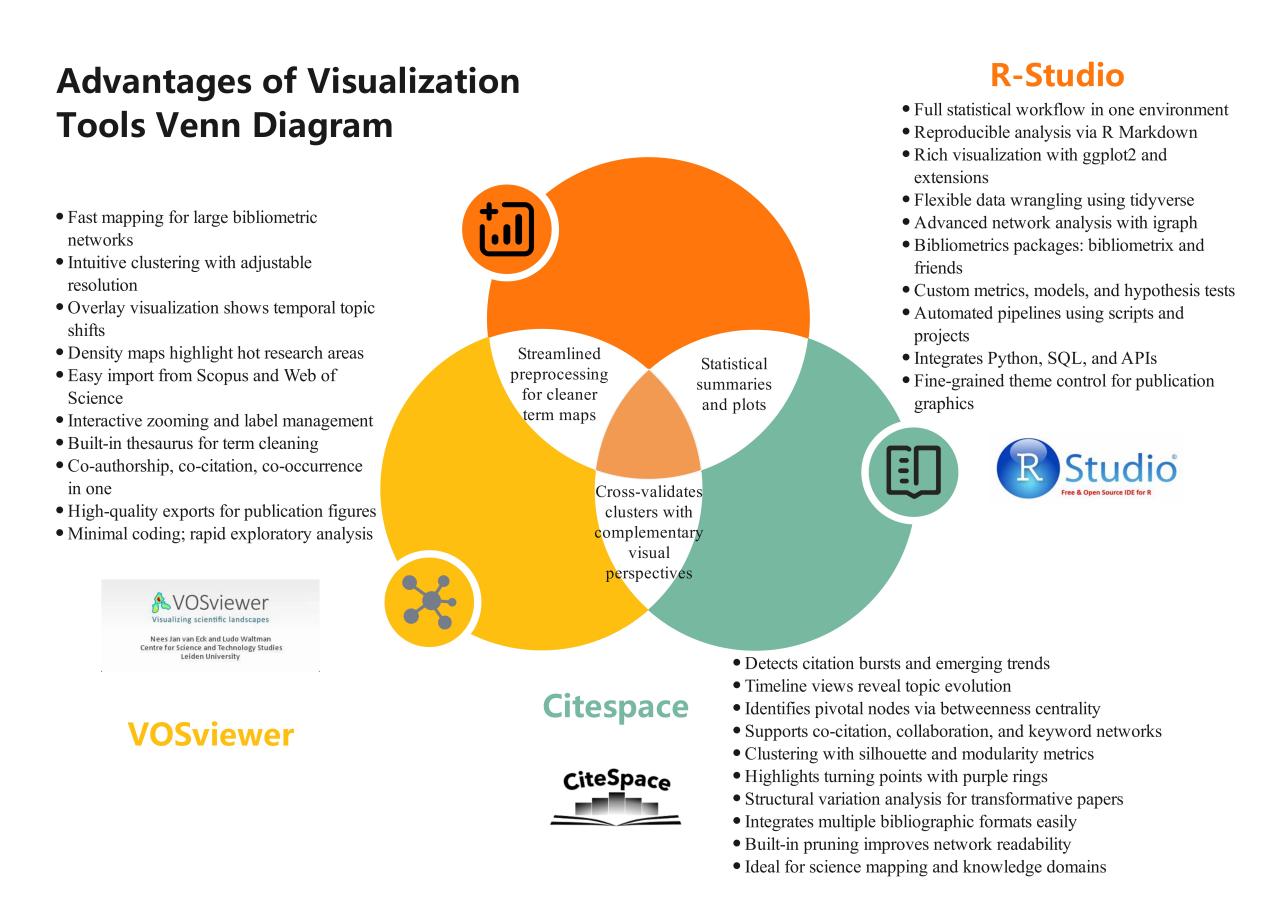 |
| --- |
| **Supplementary Material 1 Figure 1** Bibliometric commonly used software advantages Venn diagram |

**Supplementary Material 1 Table 1** Top 20 countries / Regions with the highest number of citations

| **Country / Region** | **Documents** | **Citations** | **Total Link Strength** |
| --- | --- | --- | --- |
| usa | 1640 | 148379 | 895 |
| italy | 476 | 34098 | 269 |
| The UK | 306 | 33445 | 358 |
| peoples r china | 603 | 22419 | 229 |
| germany | 268 | 21798 | 282 |
| japan | 415 | 18762 | 136 |
| australia | 214 | 17856 | 147 |
| canada | 262 | 17369 | 235 |
| south korea | 198 | 15547 | 99 |
| france | 190 | 12597 | 237 |
| spain | 209 | 10294 | 161 |
| india | 207 | 8593 | 130 |
| switzerland | 76 | 6056 | 105 |
| sweden | 80 | 5444 | 129 |
| belgium | 67 | 5239 | 77 |
| netherlands | 88 | 4789 | 126 |
| chile | 51 | 3605 | 49 |
| israel | 66 | 3245 | 64 |
| poland | 74 | 2990 | 59 |
| portugal | 50 | 2889 | 43 |

| 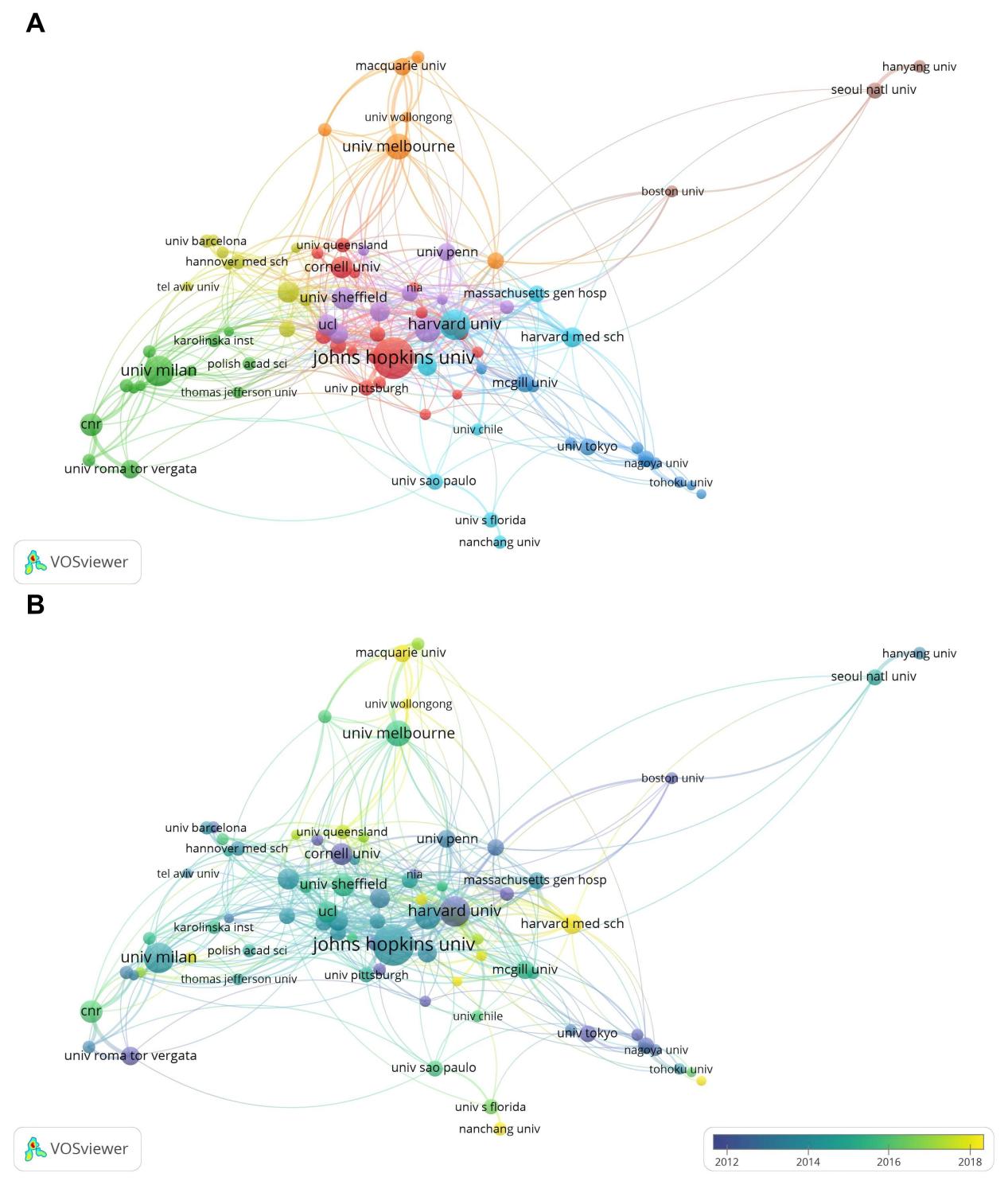 |
| --- |
| **Supplementary Material 1 Figure 2** Cluster Analysis of Publishing Institutions (The minimum threshold for the node is 20) (a) Cluster Analysis of Publishing Institutions (b) Timeline of Publication Bursts of Institutions |

**Supplementary Material 1 Table 2** Top 20 institutions with the highest number of citations

| **Organization** | **Documents** | **Citations** | **Total link strength** |
| --- | --- | --- | --- |
| johns hopkins univ | 100 | 12999 | 34 |
| cornell univ | 48 | 10984 | 21 |
| univ melbourne | 59 | 9878 | 41 |
| univ calif san diego | 56 | 9457 | 49 |
| univ calif san francisco | 33 | 8710 | 35 |
| harvard univ | 70 | 7533 | 67 |
| kings coll london | 46 | 7327 | 43 |
| columbia univ | 44 | 7207 | 41 |
| univ cambridge | 23 | 6816 | 21 |
| stanford univ | 21 | 6431 | 33 |
| univ milan | 71 | 4769 | 35 |
| univ massachusetts | 42 | 3994 | 44 |
| ohio state univ | 44 | 3990 | 17 |
| univ penn | 39 | 3748 | 26 |
| massachusetts gen hosp | 35 | 3674 | 47 |
| univ sheffield | 50 | 3548 | 31 |
| harvard med sch | 44 | 3393 | 44 |
| univ sydney | 28 | 3383 | 20 |
| emory univ | 33 | 3331 | 26 |
| ucl | 46 | 3331 | 24 |

| 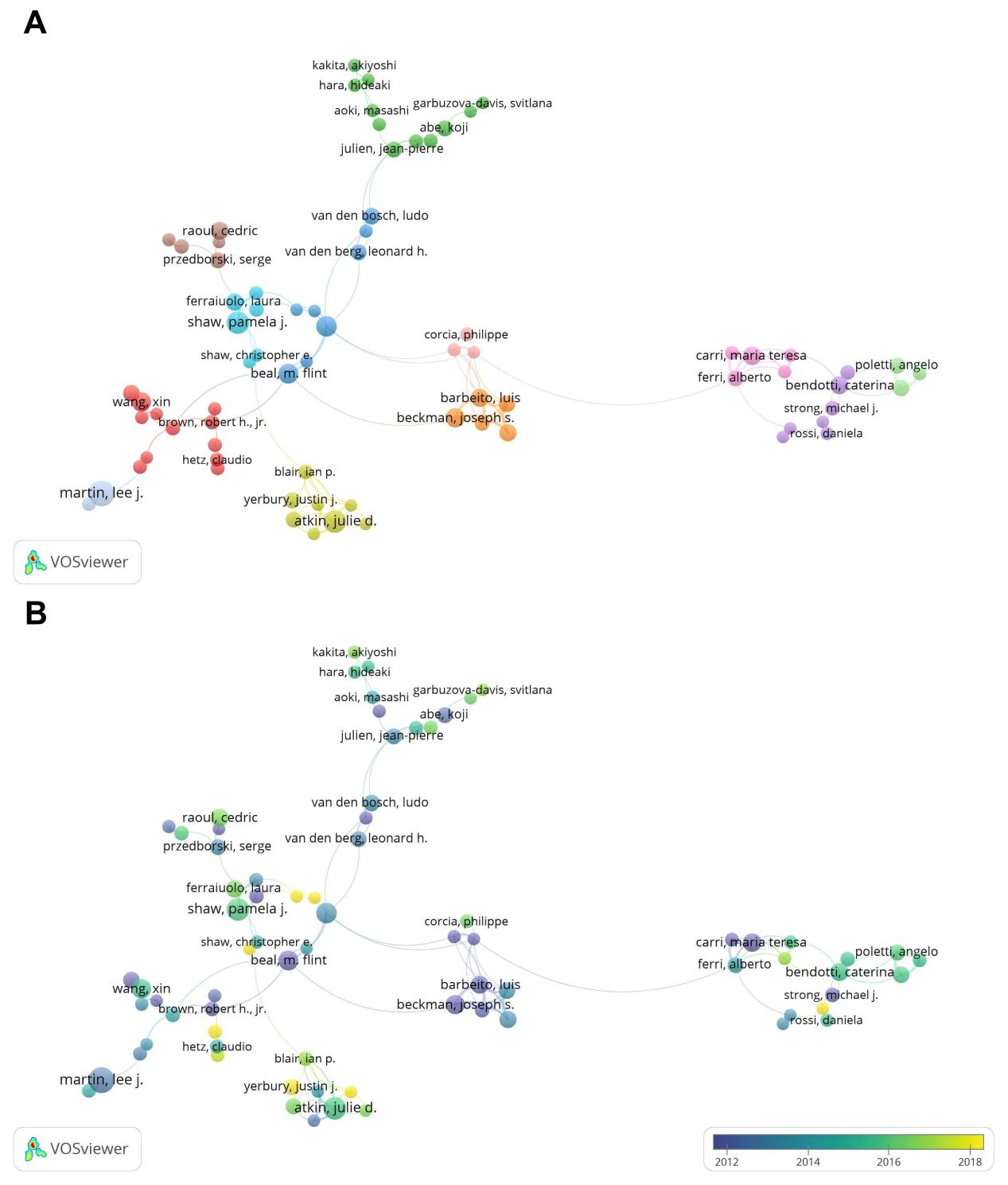 |
| --- |
| **Supplementary Material 1 Figure 3** Cluster Analysis of Publishing Authors (The minimum threshold for the node is 10) (a) Cluster Analysis of Publishing Authors (b) Timeline of Publishing Author Bursts |

**Supplementary Material 1 Table 3** Top 20 authors with the highest number of citations

| **Author** | **Documents** | **Citations** | **Total link strength** |
| --- | --- | --- | --- |
| beal, m. flint | 21 | 7365 | 11 |
| beckman, joseph s. | 20 | 5941 | 33 |
| shaw, christopher e. | 11 | 4235 | 4 |
| cleveland, don w. | 13 | 3600 | 8 |
| henderson, christopher e. | 10 | 2704 | 5 |
| martin, lee j. | 32 | 2607 | 13 |
| przedborski, serge | 14 | 2603 | 4 |
| blair, ian p. | 13 | 2429 | 17 |
| yuan, junying | 12 | 2205 | 1 |
| shaw, pamela j. | 26 | 2202 | 12 |
| kaspar, brian k. | 12 | 2196 | 13 |
| pasinelli, piera | 11 | 1911 | 2 |
| atkin, julie d. | 25 | 1666 | 37 |
| ferraiuolo, laura | 16 | 1546 | 16 |
| vargas, marcelo r. | 17 | 1537 | 31 |
| rothstein, jeffrey d. | 10 | 1533 | 5 |
| patani, rickie | 10 | 1289 | 1 |
| hetz, claudio | 12 | 1273 | 2 |
| brown, robert h., jr. | 13 | 1264 | 9 |
| barbeito, luis | 18 | 1259 | 40 |

| 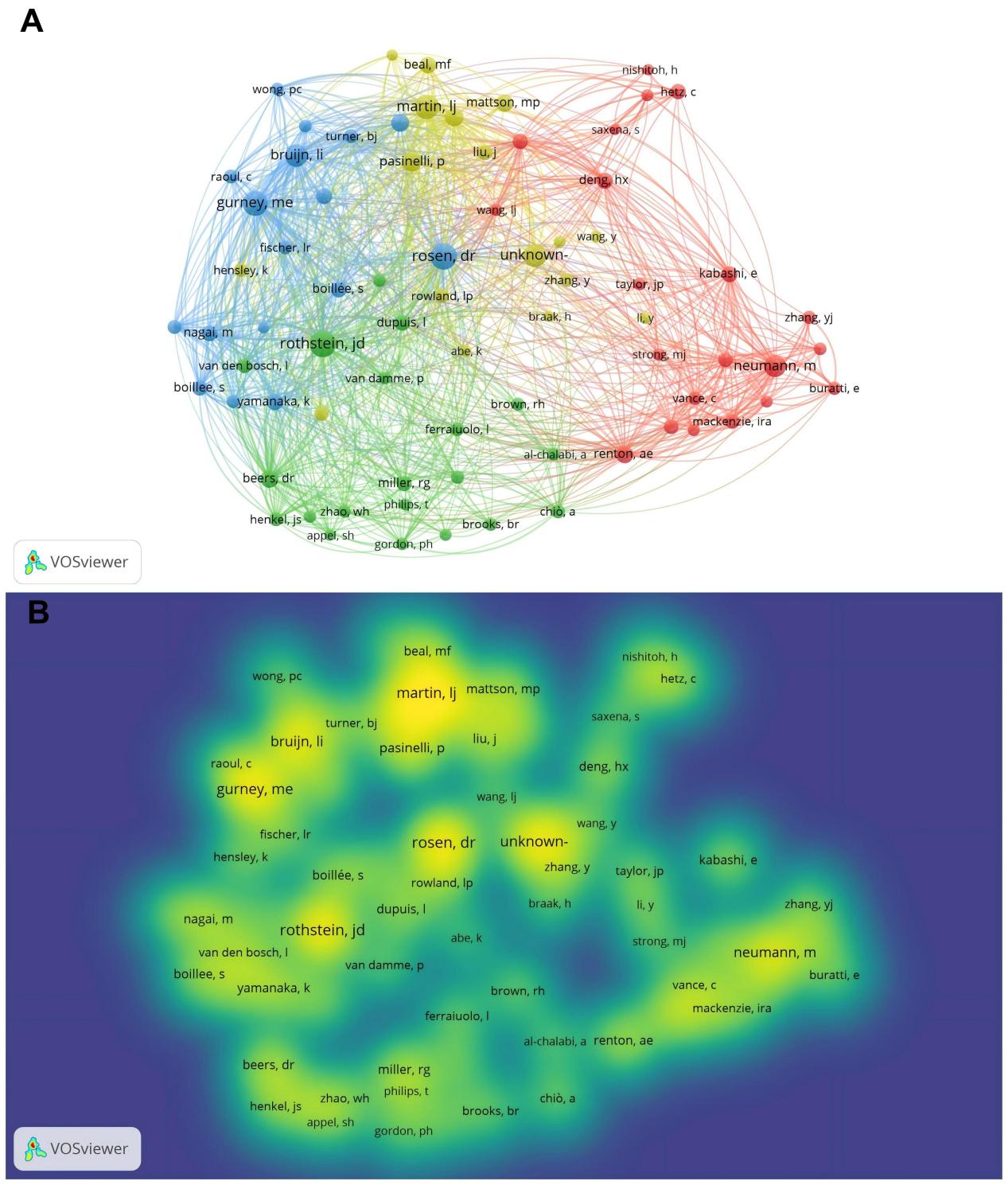 |
| --- |
| **Supplementary Material 1 Figure 4** Co-cited author clustering analysis of publications (The minimum threshold for the node is 200) (a) Co-cited author clustering analysis of publications (b) Time heatmap of co-cited authors of publishing authors |

**Supplementary Material 1 Table 4** Top 20 Co-Cited authors with the highest number of citations

| **Id** | **Author** | **Citations** | **Total link strength** |
| --- | --- | --- | --- |
| 95922 | rosen, dr | 883 | 9118 |
| 96279 | rothstein, jd | 858 | 10824 |
| 42451 | gurney, me | 809 | 8863 |
| 71506 | martin, lj | 770 | 7468 |
| 115191 | unknown- | 704 | 3990 |
| 13891 | bruijn, li | 661 | 8111 |
| 80781 | neumann, m | 623 | 6063 |
| 86413 | pasinelli, p | 542 | 6447 |
| 99176 | sasaki, s | 465 | 5988 |
| 119360 | wang, j | 452 | 4421 |
| 8441 | beers, dr | 436 | 6253 |
| 93832 | renton, ae | 421 | 4749 |
| 72589 | mattson, mp | 404 | 2422 |
| 8188 | beal, mf | 391 | 3461 |
| 124041 | yamanaka, k | 386 | 5485 |
| 79426 | nagai, m | 362 | 5020 |
| 26179 | deng, hx | 358 | 4779 |
| 54265 | kabashi, e | 352 | 4734 |
| 11487 | boillée, s | 349 | 4107 |
| 20852 | cleveland, dw | 347 | 3169 |
