# Supplementary material for: From knowledge landscapes to network mechanisms: charting regulated cell death pathways in ALS
Source: Front Aging Neurosci. 2026 Jan 30;18:1742805. doi: 10.3389/fnagi.2026.1742805 (PMC12901334; doi:10.3389/fnagi.2026.1742805)
Supplement: Supplementary file 2 [file Data_Sheet_2.docx]

**Supplementary Material 2 Retrieve records and clean records**

**1.WOSCC**

TS=(Cell death OR programmed cell death OR apoptosis OR necrosis OR Pyroptosis OR Ferroptosis OR iron death OR Copper death OR Cuproptosis OR Ammonia death OR PANoptosis) AND TS=(amyotrophic lateral sclerosis OR Lou Gehrig's disease OR progressive muscle atrophy OR ALS)


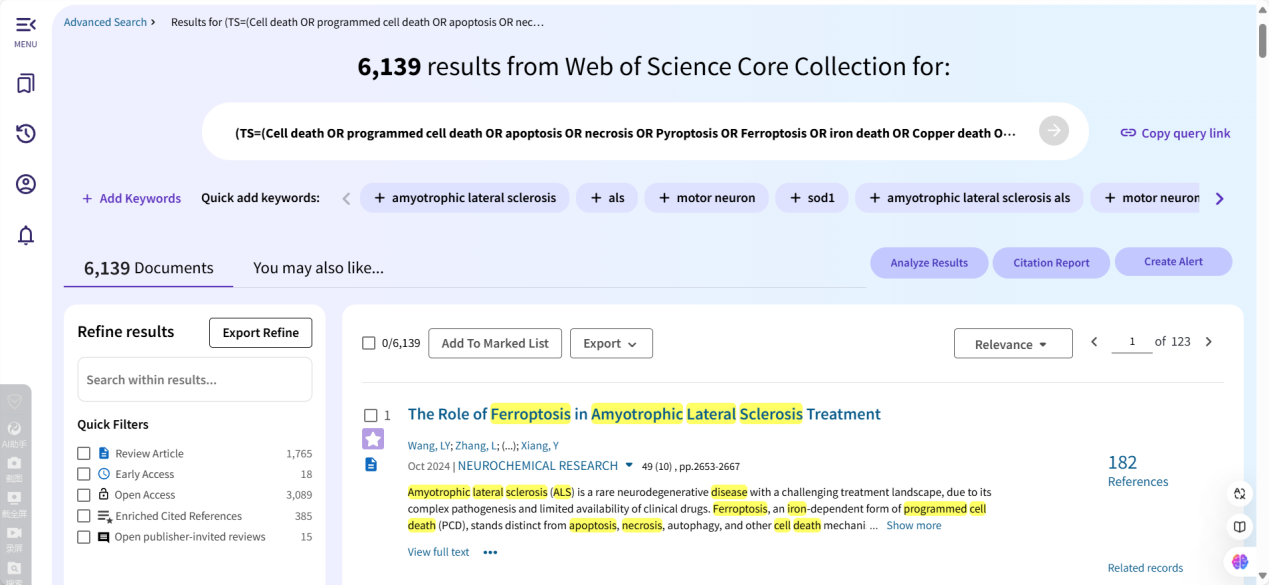


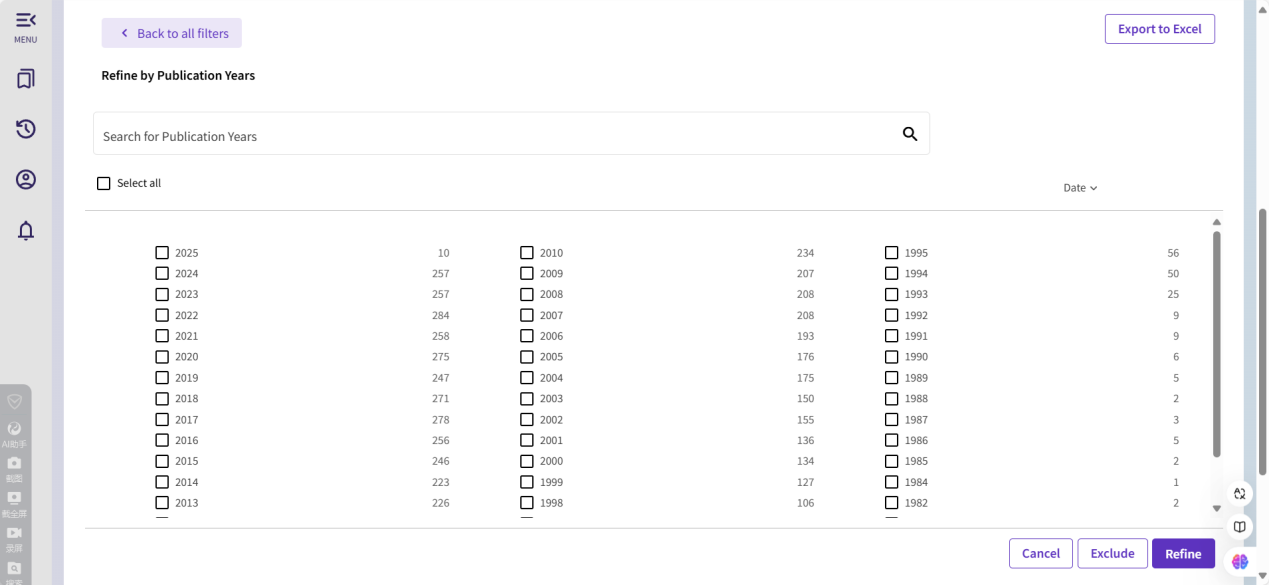


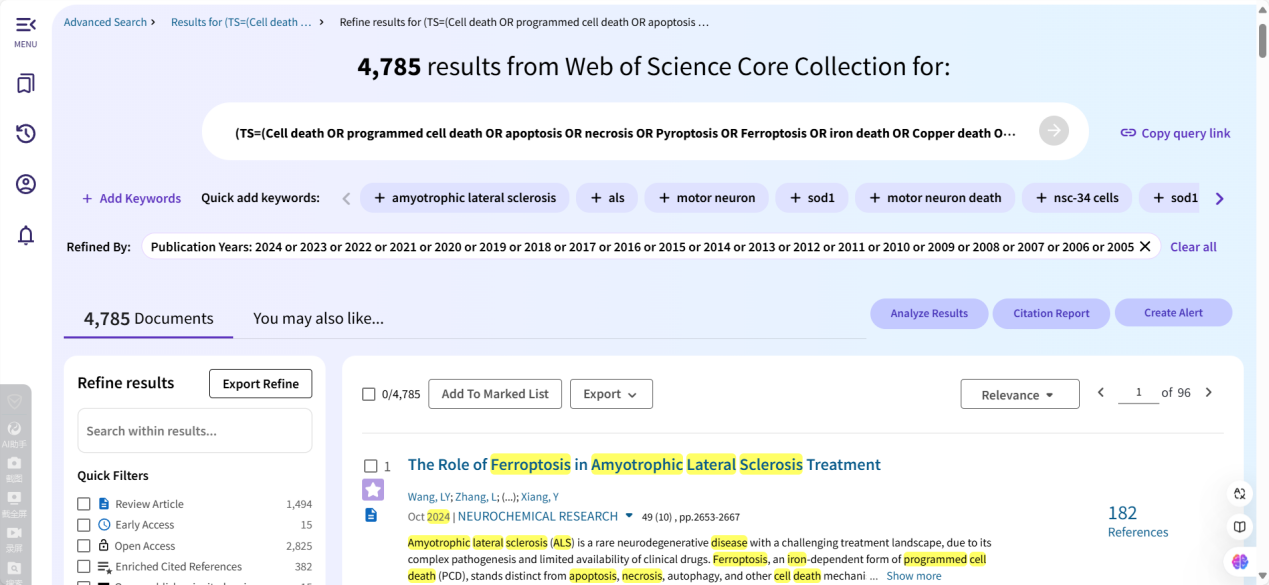

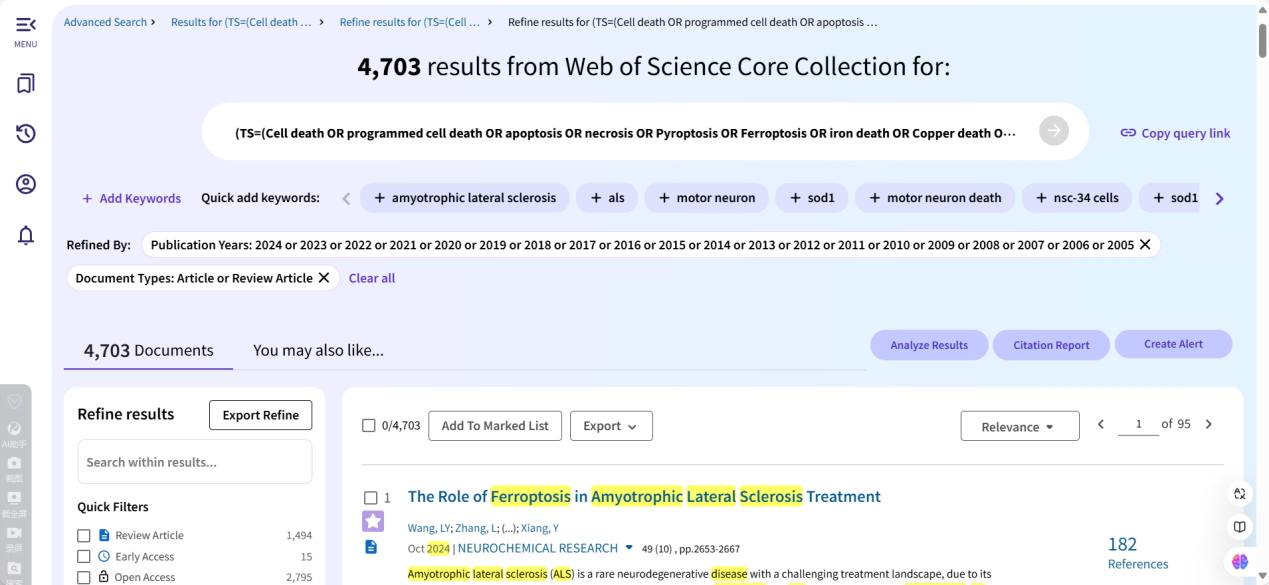


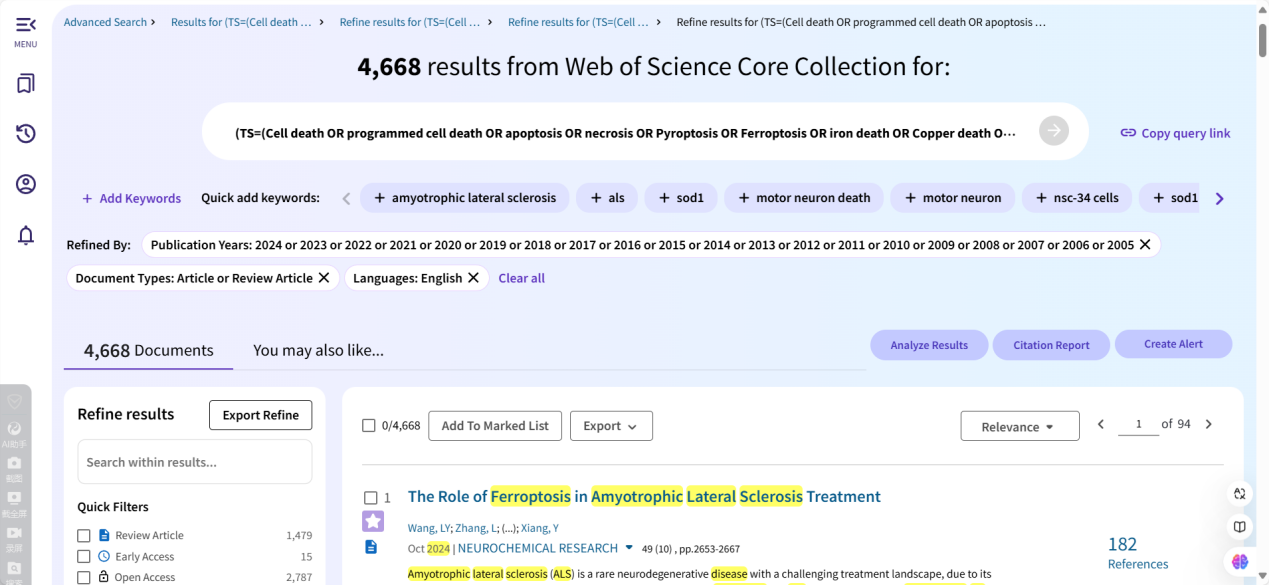


| **Records Found in Total** | 4668 |
| --- | --- |
| **Unique Records** | 4668 |
| **Duplicated Records** | 0 |
| **Invalid Records** | 0 |

**Unique Records by Source**

| 4668 | WOS |
| --- | --- |

**Document Types Retained and (Removed)**

| **3104** | Article |
| --- | --- |
| **13** | Article; Book Chapter |
| **9** | Article; Early Access |
| **55** | Article; Proceedings Paper |
| **1** | Article; Publication with Expression of Concern |
| **7** | Article; Retracted Publication |
| **1454** | Review |
| **19** | Review; Book Chapter |
| **6** | Review; Early Access |

**Statistics of Fields**

| **AB** | 4660 | 99.82862% |
| --- | --- | --- |
| **DOI** | 4668 | 100.0% |
| **PMID** | 4555 | 97.57926% |
| **DE** | 4655 | 99.72151% |
| **WC** | 4668 | 100.0% |
| **SC** | 4668 | 100.0% |
| **CR** | 4665 | 99.93573% |
| **RR** | 0 | 0.0% |
| **XX** | 0 | 0.0% |
| **YY** | 0 | 0.0% |
| **ZZ** | 0 | 0.0% |

**Results saved to: G:\Bibliometric\output**

| 2005 | 169 |
| --- | --- |
| 2006 | 185 |
| 2007 | 202 |
| 2008 | 197 |
| 2009 | 199 |
| 2010 | 227 |
| 2011 | 264 |
| 2012 | 209 |
| 2013 | 220 |
| 2014 | 213 |
| 2015 | 244 |
| 2016 | 248 |
| 2017 | 276 |
| 2018 | 263 |
| 2019 | 236 |
| 2020 | 252 |
| 2021 | 262 |
| 2022 | 260 |
| 2023 | 262 |
| 2024 | 273 |
| 2025 | 7(out) |

**Unique Entities**

| **Articles** | 4668 |
| --- | --- |
| **Journals** | 977 |
| **Authors** | 22052 |
| **Institutions** | 13970 |
| **Countries/Regions** | 612 |

Total 4661 items for analysis.

**2. Pubmed**

(Cell death OR programmed cell death OR apoptosis OR necrosis OR Pyroptosis OR Ferroptosis OR iron death OR Copper death OR Cuproptosis OR Ammonia death OR PANoptosis) AND (amyotrophic lateral sclerosis OR Lou Gehrig's disease OR progressive muscle atrophy OR ALS)


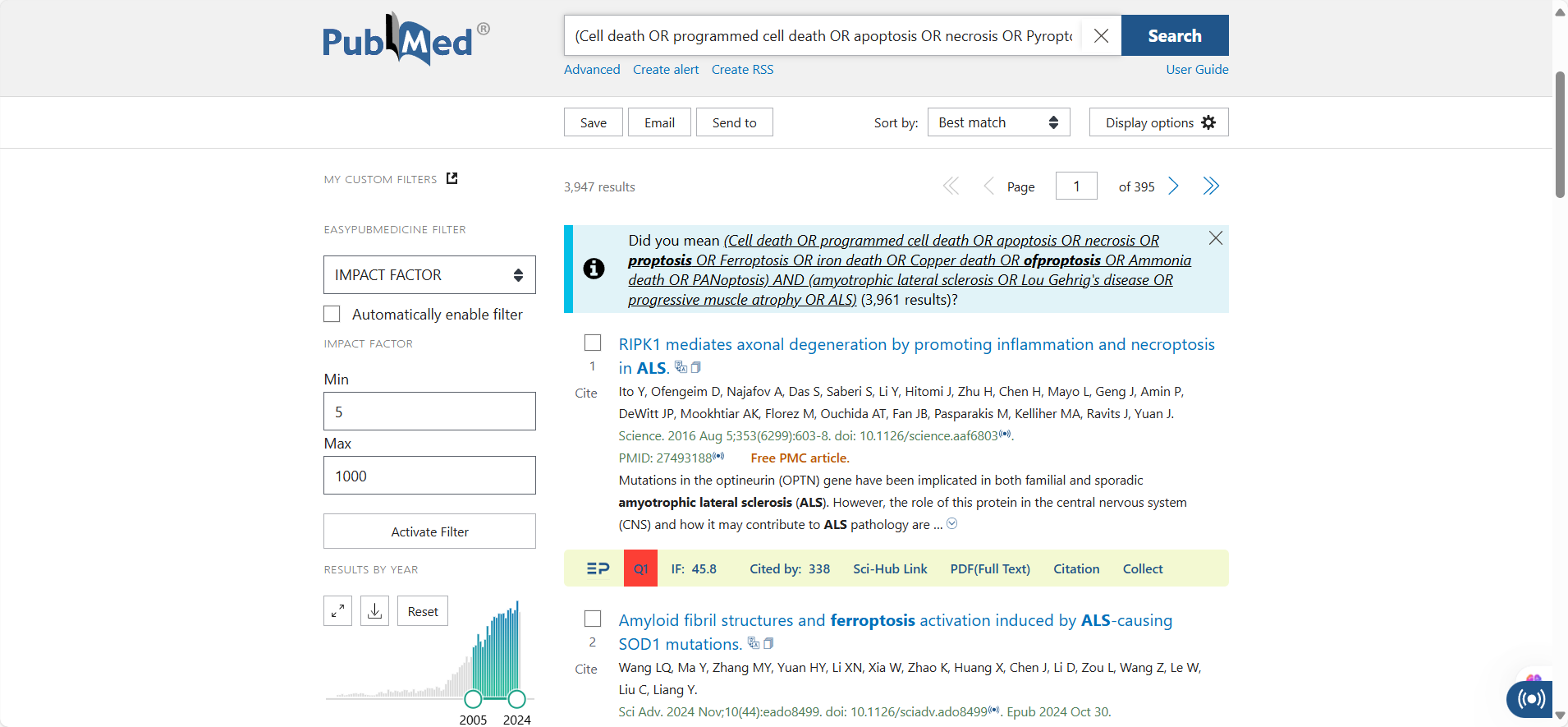


| **Records Found in Total** | 3939 |
| --- | --- |
| **Unique Records** | 1102 |
| **Duplicated Records** | 0 |
| **Invalid Records** | 0 |

**Unique Records by Source**

| 1102 | PubMed |
| --- | --- |

**Document Types Retained and (Removed)**

| **3** | Article |
| --- | --- |
| **1099** | Review |
| **(9)** | Editorial |
| **(2800)** | Journal Article |
| **(20)** | Letter |
| **(8)** | News |

**Statistics of Fields**

| **AB** | 1093 | 99.183304% |
| --- | --- | --- |
| **DOI** | 1055 | 95.73502% |
| **PMID** | 1102 | 100.0% |
| **DE** | 632 | 57.350273% |
| **WC** | 942 | 85.48094% |
| **SC** | 0 | 0.0% |
| **CR** | 0 | 0.0% |
| **RR** | 0 | 0.0% |
| **XX** | 0 | 0.0% |
| **YY** | 0 | 0.0% |
| **ZZ** | 0 | 0.0% |

**Results saved to: G:\Bibliometric\output**

| 2004 | 2舍 |
| --- | --- |
| 2005 | 31 |
| 2006 | 46 |
| 2007 | 46 |
| 2008 | 39 |
| 2009 | 44 |
| 2010 | 48 |
| 2011 | 46 |
| 2012 | 32 |
| 2013 | 53 |
| 2014 | 42 |
| 2015 | 59 |
| 2016 | 57 |
| 2017 | 53 |
| 2018 | 50 |
| 2019 | 55 |
| 2020 | 73 |
| 2021 | 86 |
| 2022 | **82** |
| 2023 | 80 |
| 2024 | 74 |
| 2025 | 4(out) |

**Unique Entities**

| **Articles** | 1102 |
| --- | --- |
| **Journals** | 455 |
| **Authors** | 3977 |
| **Institutions** | 2887 |
| **Countries/Regions** | 1099 |

Total 1093 items for analysis.

**3.Scopus**

TITLE-ABS-KEY(Cell death OR programmed cell death OR apoptosis OR necrosis OR Pyroptosis OR Ferroptosis OR iron death OR Copper death OR Cuproptosis OR Ammonia death OR PANoptosis) AND TITLE-ABS-KEY(amyotrophic lateral sclerosis OR Lou Gehrig's disease OR progressive muscle atrophy OR ALS)


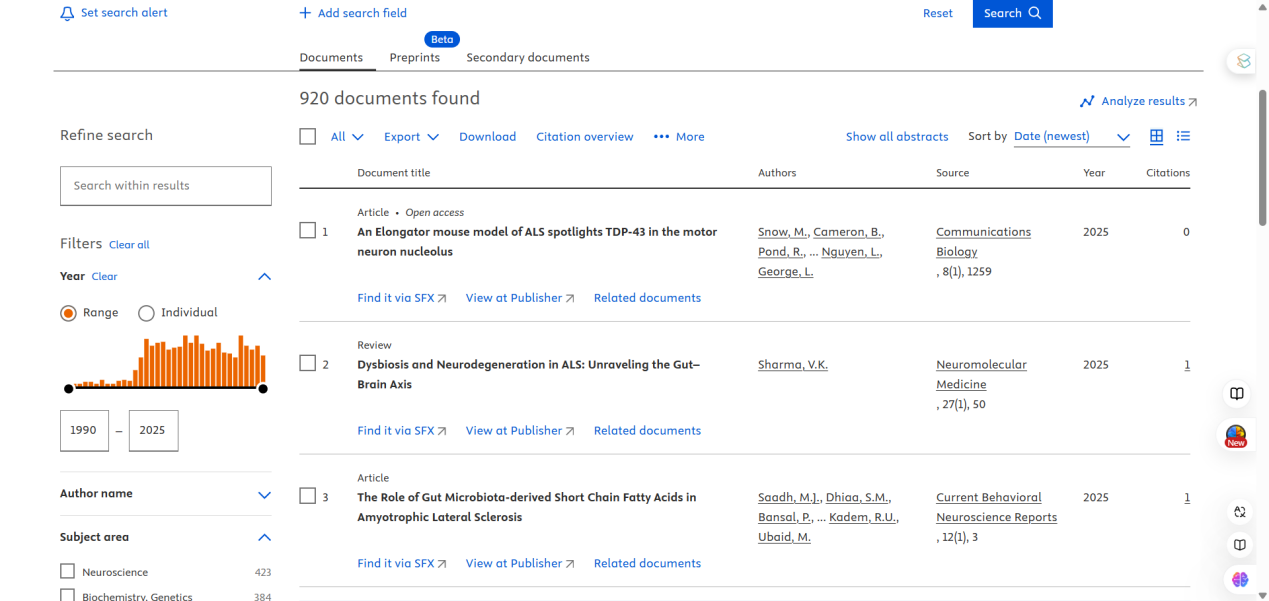


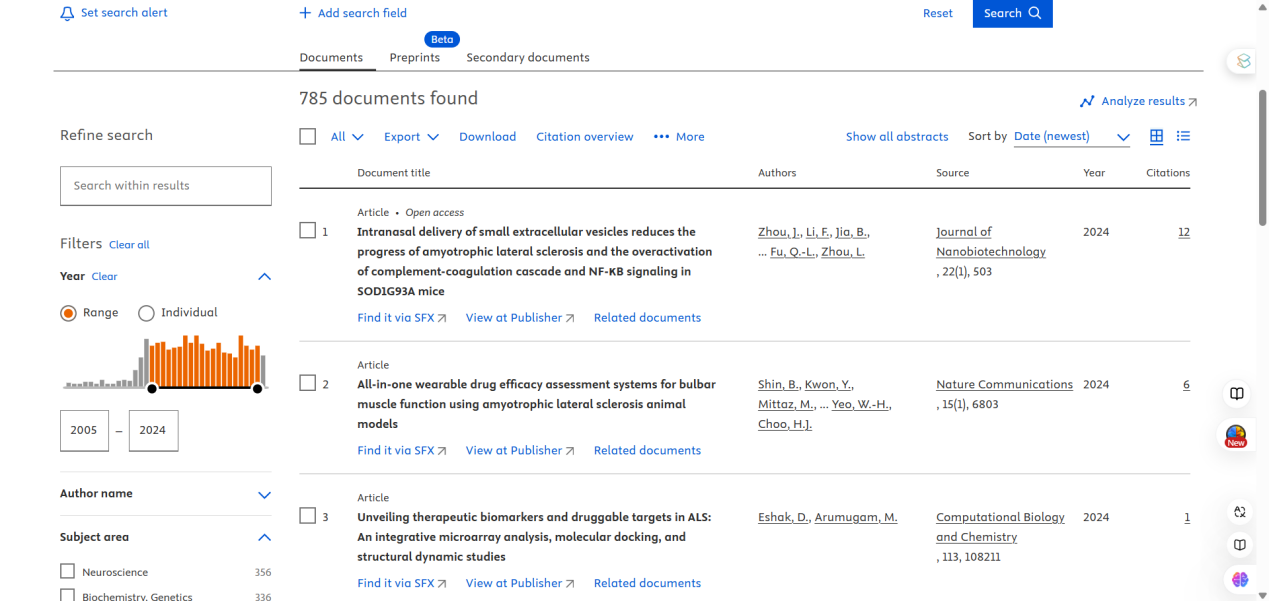


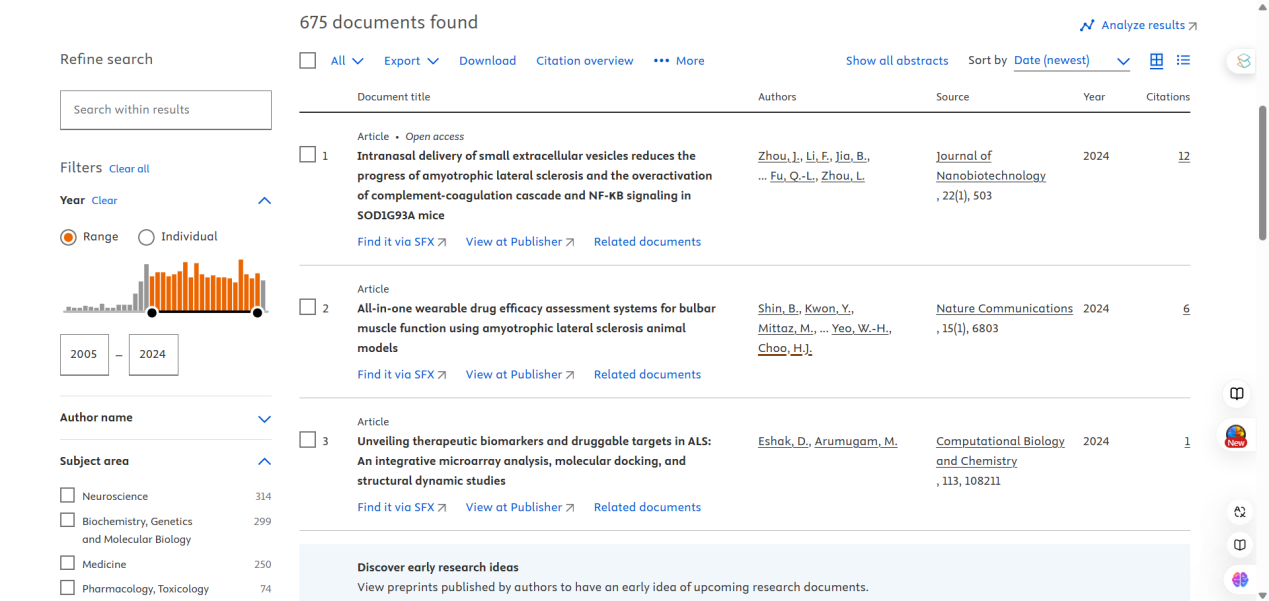


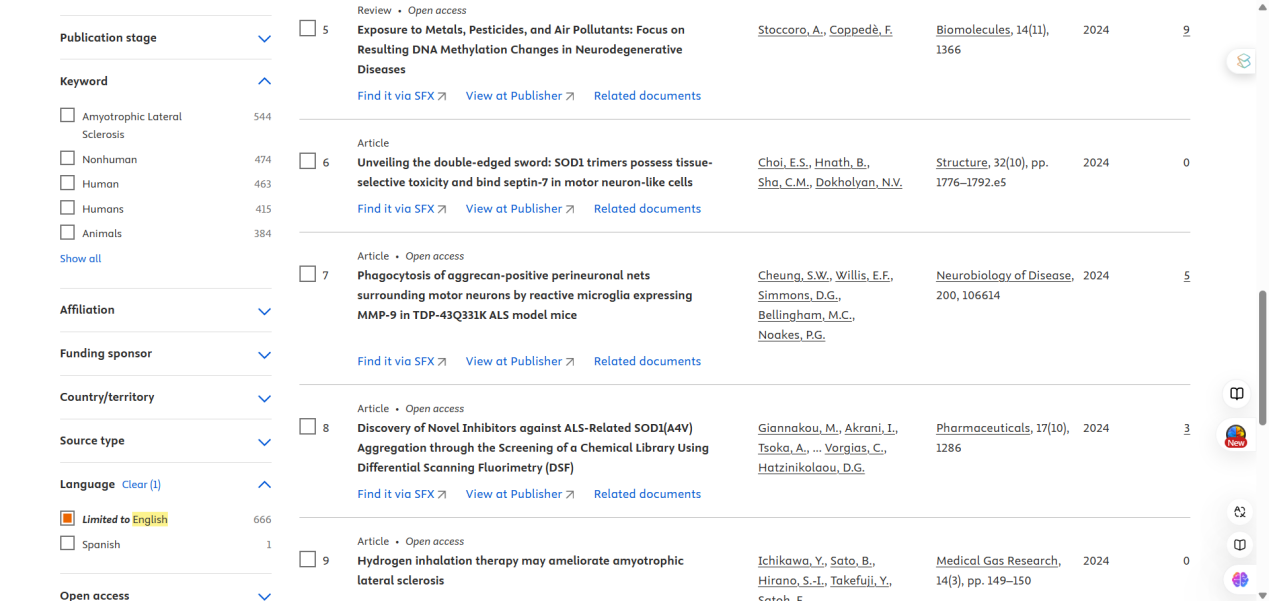


| **Records Found in Total** | 791 |
| --- | --- |
| **Unique Records** | 791 |
| **Duplicated Records** | 0 |
| **Invalid Records** | 0 |

**Unique Records by Source**

| 791 | Scopus |
| --- | --- |

**Document Types Retained and (Removed)**

| **791** | Article |
| --- | --- |

**Statistics of Fields**

| **AB** | 791 | 100.0% |
| --- | --- | --- |
| **DOI** | 784 | 99.11504% |
| **PMID** | 0 | 0.0% |
| **DE** | 780 | 98.60935% |
| **WC** | 0 | 0.0% |
| **SC** | 0 | 0.0% |
| **CR** | 790 | 99.87358% |
| **RR** | 0 | 0.0% |
| **XX** | 0 | 0.0% |
| **YY** | 0 | 0.0% |
| **ZZ** | 0 | 0.0% |

**Results saved to: G:\Bibliometric\output**

| 1990 | 1 |
| --- | --- |
| 1993 | 2 |
| 1994 | 1 |
| 1996 | 3 |
| 1999 | 3 |
| 2000 | 3 |
| 2001 | 3 |
| 2002 | 14 |
| 2003 | 26 |
| 2004 | 42 |
| 2005 | 30 |
| 2006 | 35 |
| 2007 | 33 |
| 2008 | 30 |
| 2009 | 33 |
| 2010 | 36 |
| 2011 | 44 |
| 2012 | 30 |
| 2013 | 44 |
| 2014 | 33 |
| 2015 | 29 |
| 2016 | 32 |
| 2017 | 30 |
| 2018 | 29 |
| 2019 | 28 |
| 2020 | 25 |
| 2021 | 50 |
| 2022 | 33 |
| 2023 | 29 |
| 2024 | 33 |
| 2025 | 27(out) |

**Unique Entities**

| **Articles** | 791 |
| --- | --- |
| **Journals** | 367 |
| **Authors** | 3921 |
| **Institutions** | 4038 |
| **Countries/Regions** | 84 |

Total 666 items for analysis.

**4.Combination**

| **Records Found in Total** | 6423 |
| --- | --- |
| **Unique Records** | 6382 |
| **Duplicated Records** | 41 |
| **Invalid Records** | 0 |

**Unique Records by Source**

| 661 | Scopus |
| --- | --- |
| 4661 | WOS |
| 1060 | PubMed |

**Document Types Retained and (Removed)**

| **3762** | Article |
| --- | --- |
| **13** | Article; Book Chapter |
| **9** | Article; Early Access |
| **55** | Article; Proceedings Paper |
| **1** | Article; Publication with Expression of Concern |
| **7** | Article; Retracted Publication |
| **2510** | Review |
| **19** | Review; Book Chapter |
| **6** | Review; Early Access |

**Statistics of Fields**

| **AB** | 6365 | 99.73363% |
| --- | --- | --- |
| **DOI** | 6332 | 99.216545% |
| **PMID** | 5608 | 87.87214% |
| **DE** | 5916 | 92.69821% |
| **WC** | 5569 | 87.26105% |
| **SC** | 4661 | 73.03353% |
| **CR** | 5319 | 83.34378% |
| **RR** | 0 | 0.0% |
| **XX** | 0 | 0.0% |
| **YY** | 0 | 0.0% |
| **ZZ** | 0 | 0.0% |

**Results saved to: G:\Bibliometric\output**

| 2005 | 228 |
| --- | --- |
| 2006 | 263 |
| 2007 | 278 |
| 2008 | 264 |
| 2009 | 274 |
| 2010 | 310 |
| 2011 | 352 |
| 2012 | 268 |
| 2013 | 314 |
| 2014 | 287 |
| 2015 | 330 |
| 2016 | 334 |
| 2017 | 357 |
| 2018 | 339 |
| 2019 | 318 |
| 2020 | 349 |
| 2021 | 397 |
| 2022 | 372 |
| 2023 | 370 |
| 2024 | 378 |

**Unique Entities**

| **Articles** | 6382 |
| --- | --- |
| **Journals** | 1729 |
| **Authors** | 26269 |
| **Institutions** | 20144 |
| **Countries/Regions** | 1657 |

Log file: G:\Bibliometric\output\log.txt
